# An EGF-like Protein Forms a Complex with PfRh5 and Is Required for Invasion of Human Erythrocytes by *Plasmodium falciparum*

**DOI:** 10.1371/journal.ppat.1002199

**Published:** 2011-09-01

**Authors:** Lin Chen, Sash Lopaticki, David T. Riglar, Chaitali Dekiwadia, Alex D. Uboldi, Wai-Hong Tham, Matthew T. O'Neill, Dave Richard, Jake Baum, Stuart A. Ralph, Alan F. Cowman

**Affiliations:** 1 The Walter and Eliza Hall Institute of Medical Research, Melbourne, Australia; 2 Department of Medical Biology, University of Melbourne, Melbourne, Australia; 3 Department of Biochemistry and Molecular Biology, Bio21 Molecular Sciences and Biotechnology Institute, University of Melbourne, Melbourne, Australia; MRC National Institute for Medical Research, United Kingdom

## Abstract

Invasion of erythrocytes by *Plasmodium falciparum* involves a complex cascade of protein-protein interactions between parasite ligands and host receptors. The reticulocyte binding-like homologue (PfRh) protein family is involved in binding to and initiating entry of the invasive merozoite into erythrocytes. An important member of this family is PfRh5. Using ion-exchange chromatography, immunoprecipitation and mass spectroscopy, we have identified a novel cysteine-rich protein we have called *P. falciparum* Rh5 interacting protein (PfRipr) (PFC1045c), which forms a complex with PfRh5 in merozoites. Mature PfRipr has a molecular weight of 123 kDa with 10 epidermal growth factor-like domains and 87 cysteine residues distributed along the protein. In mature schizont stages this protein is processed into two polypeptides that associate and form a complex with PfRh5. The PfRipr protein localises to the apical end of the merozoites in micronemes whilst PfRh5 is contained within rhoptries and both are released during invasion when they form a complex that is shed into the culture supernatant. Antibodies to PfRipr1 potently inhibit merozoite attachment and invasion into human red blood cells consistent with this complex playing an essential role in this process.

## Author Summary

The malaria parasite invades red blood cells by binding to proteins on the surface of this host cell. A family of proteins called *P. falciparum* reticulocyte binding-like homologue (PfRh) proteins are important for recognition of the red blood cell and activation of the invasion process. An important member of the PfRh family is PfRh5. We have identified a novel cysteine-rich protein we have called *P. falciparum* Rh5 interacting protein (PfRipr), which forms a complex with PfRh5 in merozoites. PfRipr has 10 epidermal growth factor-like domains and is expressed in mature schizont stages where it is processed into two polypeptides that associate and form a complex with PfRh5. The PfRipr protein localises to the apical end of the merozoites in micronemes whilst PfRh5 is contained within rhoptries and both are released during invasion when they form a complex that is released into the culture supernatant. Antibodies to PfRipr1 can potently inhibit merozoite attachment and invasion into human red blood cells consistent with this complex playing an essential role in this process.

## Introduction

Malaria is caused by parasites from the genus *Plasmodium*, of which *Plasmodium falciparum* is associated with the most severe form of the disease in humans. Sporozoite forms of these parasites are injected into humans during mosquito feeding and they migrate to the liver where they invade hepatocytes and develop into merozoites, which are released to invade erythrocytes in the blood stream. The blood stage cycle of *P. falciparum* is responsible for all of the clinical symptoms associated with malaria [1]. Once a merozoite has invaded an erythrocyte it develops, within this protected intracellular niche, to form around 16 new merozoites that are released and then bind and invade other red blood cells. Invasion of merozoites into the host erythrocyte is a rapid process involving multiple steps in a cascade of protein-protein interactions (see for review [2]).

The reticulocyte binding-like homologues (PfRh or PfRBP) and erythrocyte binding-like (EBL) proteins play important roles in merozoite invasion [3], [4], [5], [6], [7], [8], [9], [10], [11], [12], [13], [14], [15]. The PfRh family consists of PfRh1 (PFD0110w), PfRh2a (PF13_0198), PfRh2b (MAL13P1.176), PfRh3 (PFL2520w), PfRh4 (PFD1150c) and PfRh5 (PFD1145c) [4], [5], [7], [9], [16], [17], [18], [19], [20]. PfRh3 is a transcribed psuedogene in all the *P. falciparum* strains that have been analysed [21]. PfRh1, PfRh2b, PfRh2a, PfRh4 and PfRh5 bind to erythrocytes and antibodies to them can inhibit merozoite invasion thus showing they play a role in this process [11], [13], [18], [19], [20], [22], [23], [24].

Polymorphisms in the PfRh5 protein have been linked to differential virulence in infection of Aotus monkeys suggesting that amino acid changes in its binding domain can switch receptor recognition [19]. PfRh5 has been shown to bind red blood cells but its putative receptor has not been identified [18], [19], [20]. In contrast to other members of the PfRh protein family, PfRh5 is considerably smaller and lacks a transmembrane region, which combined with its role as an invasion ligand, suggests it may be part of a functional complex. It has not been possible to genetically disrupt the gene encoding PfRh5 and antibodies to it can partially inhibit merozoite invasion, pointing to an essential role of this protein in the invasion process [20].

The EBL family of proteins includes EBA-175 (MAL7P1.176) [3], [26], EBA-181 (also known as JESEBL) (PFA0125c) [27], [28], EBA-140 (also known as BAEBL) (MAL13P1.60) [6], [29], [30], [31] and EBL-1 [32]. Whilst these parasite ligands function in merozoite invasion by binding to specific receptors on the erythrocyte, they appear to have a central role in activation of the invasion process. For example, it has been shown that binding of EBA-175 to its receptor, glycophorin A restores the basal cytosolic calcium levels after interaction of the merozoite with the erythrocyte and triggers release of rhoptry proteins and it is likely that the PfRh protein family plays a similar role [37].

The PfRh and EBL protein families are responsible for a mechanism of phenotypic variation that allows different strains of *P. falciparum* to invade erythrocytes using different patterns of host receptors [7], [28], [38]. This is important for evasion of host immune responses and also provides a means to circumvent the polymorphic nature of the erythrocyte surface in the human population [39]. Indeed, analysis of immune responses from individuals in malaria endemic areas has suggested that the PfRh proteins are targets of human invasion inhibitory antibodies and therefore a critical component of acquired protective immunity [39], [40], [41].

In order to understand the function of PfRh5 in merozoite invasion, we purified it from parasite culture supernatant and show this protein exists as a complex with a cysteine-rich protein containing 10 epidermal growth factor-like domains (PFC1045c). This novel protein functions with PfRh5 to play an essential role in invasion of merozoites into human erythrocytes.

## Results

### Purification and identification of a PfRh5 complex

**Figure 1:**
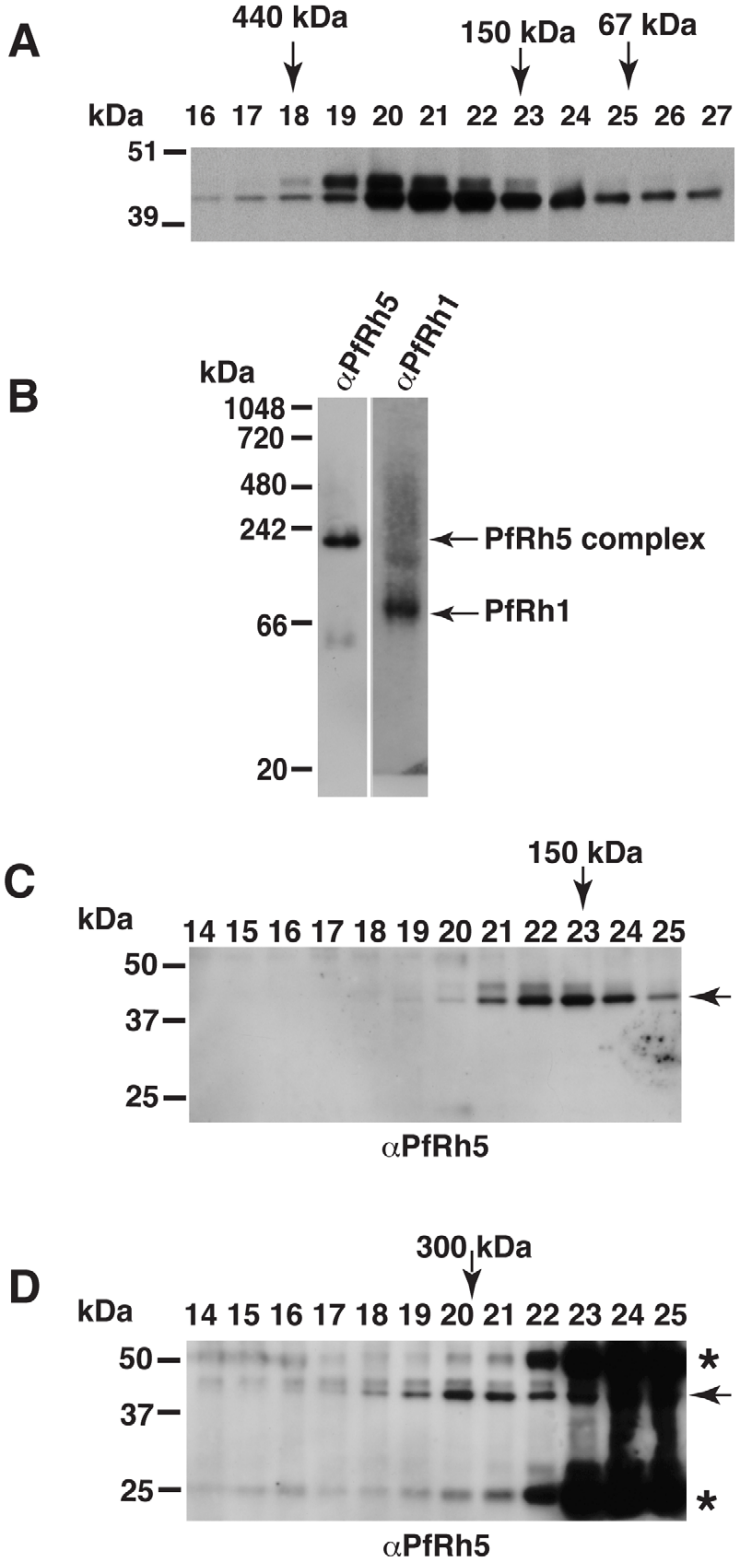
The processed 45 kDa PfRh5 C-terminal domain exists as a larger complex. (A) PfRh5 partially purified from parasite culture supernatant was analysed by size exclusion chromatography on a Superdex 200 analytical column. PfRh5 was eluted from the column as a ∼150–200 kDa species. Molecular weight of eluted protein fractions are indicated by arrows as shown using standard proteins of known size. PfRh5 was detected using 2F1, a specific monoclonal antibody to the protein. (B) Blue native gel electrophoresis confirmed that PfRh5 migrates as a ∼150–200 kDa species. The processed PfRh1 fragment of 110 kDa is included as a control. (C) 300 µl of the PfRh5-containing fraction, isolated from culture supernatant, was loaded onto a Superdex 200 analytical column and eluted with PBS. (D) An identical 300 µl sample was pre-incubated with 25 µg of monoclonal anti-PfRh5 antibody before loading onto the same Superdex 200 column. The peak of the eluted PfRh5 protein lies at two preceding fractions as compared to panel C, corresponding to an increase in molecular weight of ∼150 kDa indicating that one antibody molecule was bound to the PfRh5-containing species. The monoclonal antibody-bound PfRh5 is indicated by an arrow. PfRh5 and the immunoglobulin heavy and light chain of antibody seen in fraction #22–25 are the free PfRh5 complex and the excess antibody. The asterisks refer to cross-hybridising bands corresponding to the immunoglobulin heavy and light chain.

PfRh5 binds to erythrocytes and is functionally important in merozoite invasion of erythrocytes [18], [19], [20]. Whilst other members of the PfRh family also bind erythrocytes, they have a transmembrane region, a feature that PfRh5 lacks suggesting that it may exist as a complex. In order to determine if PfRh5 was shed as a complex into culture supernatant during merozoite invasion we set out to purify it using ion-exchange chromatography. PfRh5, that had been partially purified from culture supernatant by ion-exchange chromatography, migrated on SDS-PAGE gels as an approximately 45 kDa fragment as detected using 2F1, a anti-PfRh5 specific monoclonal antibody (data not shown), consistent with our previous results showing that the 63 kDa mature PfRh5 was processed in late schizonts and shed into culture supernatant during merozoite invasion [20]. However, analysis of PfRh5 by gel-filtration chromatography revealed that the 45 kDa polypeptide was eluted in the peak (# 19–24) (Fig. 1A) which is immediately before the peak of 158 kDa bovine Υ-globulin (#21–25) (Fig. S1), suggesting PfRh5 is indeed in a complex with a molecular weight of approximately 150–200 kDa.

To confirm PfRh5 exists as a higher molecular weight species, blue native gel electrophoresis was performed and showed the PfRh5 migrated at an approximate molecular weight of 150–200 kDa (Fig. 1 B). This was in contrast with the processed PfRh1 fragment that migrated at the expected size of 110 kDa (Fig. 1B) [12]. This suggested that PfRh5 either forms a homo-oligomer or was in complex with other molecule/s. To distinguish between these possibilities, gel-filtration chromatography was used to identify the size increment of PfRh5-containing species after it was incubated with a monoclonal anti-PfRh5 antibody [20]. It was found that pre-incubation with anti-PfRh5 antibody caused the PfRh5 protein peak to appear two fractions earlier, which correspond to an increase in size of the PfRh5-containing species by 150 kDa, indicating that one antibody molecule was associated with the complex (Fig. 1C and D). The free PfRh5 co-eluted with the excess antibody in the protein peak comprising fraction 22 to 25 (Fig. 1D), further indicating PfRh5 is in a complex of approximate 150 kDa. Therefore we proposed that the processed 45 kDa PfRh5 species formed a complex with another molecule/s rather than existing as a homo-oligomer.

To identify the proteins present within the PfRh5 complex, we performed a large-scale purification using culture supernatant from the parasite line 3D7-Rh5HA in which the *PfRh5* gene had been tagged with haemagglutinin epitopes [20]. The PfRh5 protein was enriched and partially purified using ion-exchange chromatography followed by further purification on an anti-HA affinity matrix. The bound proteins were eluted and subjected to trypsin digestion followed by analysis of the resulting peptides by mass spectrometry (LC-MS/MS) [42]. Since powerful anion ion-exchange chromatography was employed to enrich the complex and remove the majority of contaminants before it was subjected to anti-HA antibody affinity pull-down), the mass spectrometry analysis yielded a very clean result (Table S1). Database analysis identified parasite protein peptides that were predominantly derived from two proteins, which included PfRh5 and a conserved hypothetical protein (PFC1045c), designated here as *P. falciparum* PfRh5 interacting protein (PfRipr) (Table 1 and Table S1). The most N-terminal peptide found for PfRh5 was amino acid 187–197, consistent with it being shed into culture supernatant as an approximately 45 kDa fragment produced by cleavage between amino acid 125 and 135 of the mature protein. In the case of PfRipr, we identified peptides from both N-terminal and C-terminal regions of the protein suggesting that the full-length protein was present within the complex and this was confirmed by co-immunoprecipitation experiments with specific antibodies (see below section). The mature form of PfRipr was 123 kDa and this together with the 45 kDa PfRh5 fragment would produce a complex with an overall molecular weight of approximately 170 kDa in agreement with the size observed by gel-filtration chromatography and blue native gel electrophoresis (Fig. 1A and B). The PfRh5/PfRipr complex was stably purified by ion-exchange chromatography and 350 mM NaCl in the elution buffer did not disrupt their association, suggesting the tight interaction of two proteins. Such specific high affinity protein-protein interaction implies the biological significance of the complex.

**Table 1:** Peptides identified by Mass spectrometry from purified PfRh5.

Protein name	PlasmoDB Gene number	Peptide position	Peptide sequence
PfRh5	PFD1145c	187–197	(K)HLSYNSIYHK(S)
		212–221	(K)KINETYDKVK(S)
		237–247	(K)KLEHPYDINNK(N)
		303–310	(K)MMDEYNTK(K)
		358–366	(R)YHYDEYIHK(L)
		437–443	(K)IIQDKIK(L)
PfRipr	PFC1045c	93–100	(K)ScDYFISK(E)
		101–114	(K)EYNSSDKTNQIcYK(K)
		699–708	(K)LIcQcEEGYK(N)
		760–769	(K)MEDGINcIAK(N)
		963–972	(K)INcTcKENYK(N)

### PfRh5 and PfRipr form a complex in *P. falciparum*

**Figure 2:**
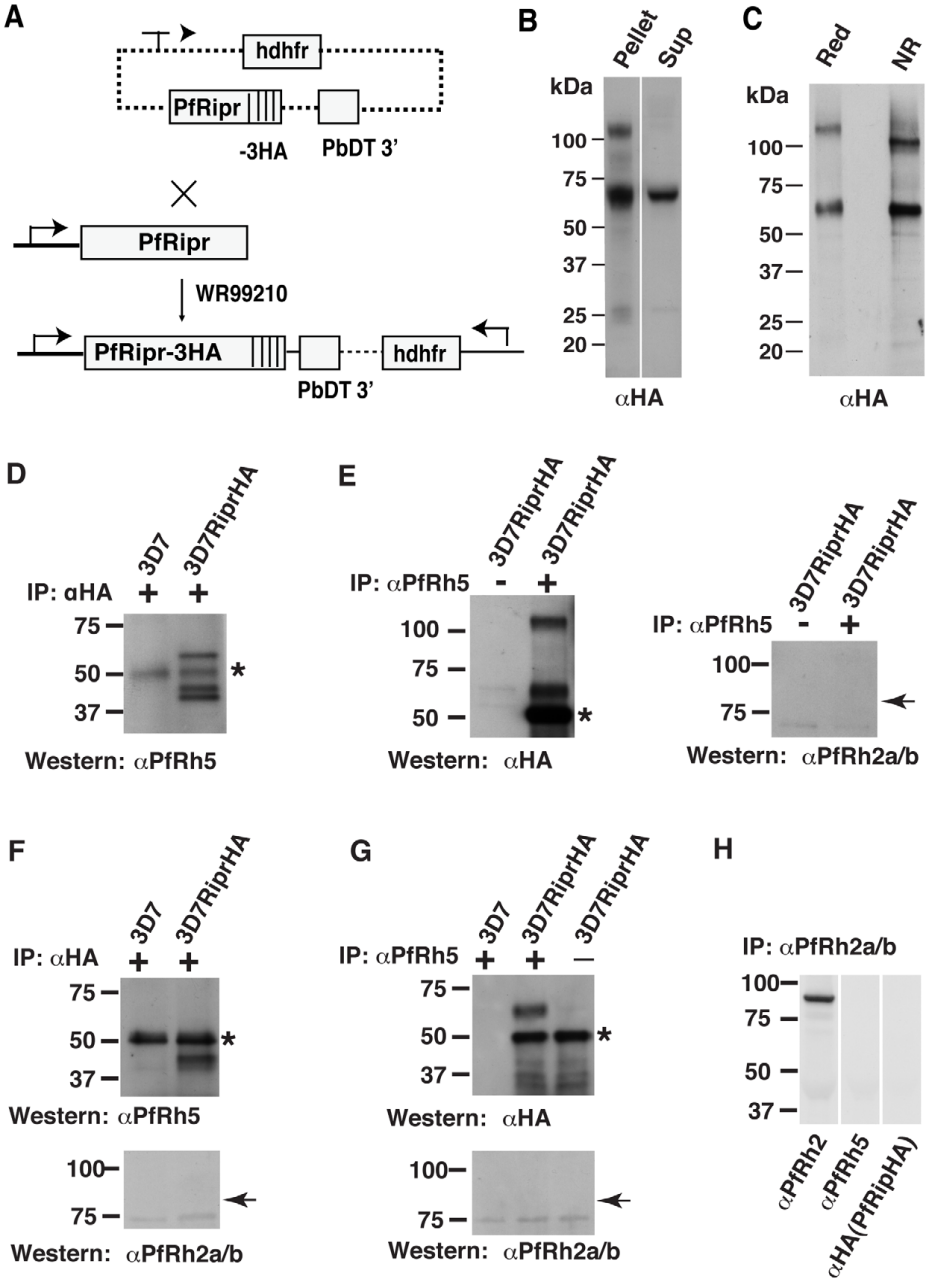
PfRh5 and PfRipr form a complex. (A) A triple Haemaglutinin (HA) tag was added to the C-terminus of PfRipr by 3′ single homologous crossover recombination. (B) HA-tagged PfRiprHA was detected in the parasite membrane pellet and in the culture supernatant of 3D7PfRiprHA line, confirming successful tagging. (C) PfRiprHA was analysed by SDS-PAGE under reducing and non-reducing conditions. Similar mobility, of the 65 kDa C-terminal fragment under both conditions, indicates that the N-terminal and C-terminal of PfRipr was not linked by disulphide bonds after processing. (D) Co-immunoprecitation of PfRh5 with PfRiprHA was performed using proteins solubilized from saponin pellets of 3D7-PfRiprHA schizont-infected erythrocytes. Immunoprecipitation of HA-tagged PfRipr by anti-HA antibody co-immunoprecipitated PfRh5. (E) Immunoprecitation with anti-PfRh5 antibody using the same schizont materials co-immunoprecipited PfRiprHA but not PfRh2a/b. (F) Co-immnuoprecipitation of PfRh5 with PfRiprHA from culture supernatants using anti-HA-Sepharose bead. Detection of PfRh5 but not PfRh2a or PfRh2b in the anti-HA bead bound material confirmed that PfRh5 was specifically co-immunoprecipitated with PfRiprHA. (G) Co-immunoprecipitation of PfRiprHA with PfRh5 from culture supernatants using monoclonal anti-PfRh5 antibody coupled to Mini-beads. Probing of the bound material with anti-HA antibody detected PfRiprHA only from the 3D7-PfRiprHA parasites and no PfRh2a/b was detected in the bound materials, indicating that PfRiprHA was specifically co-immunoprecipitated with PfRh5. (H) Immnunoprecipitation of culture supernatant from PfRiprHA parasites with rabbit anti-PfRh2a/b antibodies that recognize 85 kDa processed PfRh2a/b fragment did not pull-down PfRh5 or PfRipr. In all cases, the asterisk indicates a cross-hybridizing band corresponding to the heavy chain of IgG eluted from the antibody affinity beads. The arrows in Figure 2E, F and G indicate where 85 kDa PfRh2a/b band should appear.

In order to confirm that PfRh5 and PfRipr form a complex, we inserted a plasmid by single cross-over homologous recombination at the 3′ end of the *Pfripr* gene to fuse three HA epitopes and derived the parasite line 3D7RiprHA (Fig. 2A) [20]. Successful tagging was confirmed by immunoblot experiments using anti-HA antibodies on both saponin lysed schizont pellet preparations and proteins from culture supernatants (Fig. 2B). In parasite schizont pellets a minor band of approximately 125 kDa and a major band of approximately 65 kDa were detected whereas in culture supernatants only the smaller protein band was predominantly observed. The 125 kDa protein presumably represents the full-length mature protein in schizonts that was processed and shed into the culture supernatant. Since only one processed fragment of 65 kDa was detected in culture supernatants using anti-HA antibody, we propose that PfRipr was processed into at least two fragments of approximately the same size. Mobility of the 65 kDa fragment remains similar under the reducing and non-reducing condition (Fig. 2C) demonstrating that the processed fragments are not linked by a disulphide bond. Both 65 and 125 kDa fragments migrated slightly faster on SDS-PAGE gels under non-reducing conditions consistent with the presence of multiple intramolecular disulfide linkages creating tightly folded domains (Fig. 2C).

To further confirm that PfRh5 and PfRipr form a complex, we performed co-immunoprecipitation experiments with anti-PfRh5 and anti-HA antibodies using proteins from the parasite line 3D7RiprHA (Fig. 2D to G). Immunoprecipitation with anti-HA antibodies, to pull-down tagged PfRipr from schizont-infected erythrocytes, also brought down PfRh5 as shown by immunoblots with a specific monoclonal anti-PfRh5 antibody (Fig. 2D). The full-length mature protein (approximately 63 kDa) as well as two processed fragments of similar molecular weight (approximately 45 kDa) was observed (Fig. 2D). The reciprocal experiment in which we immunoprecipitated PfRh5, using the monoclonal anti-PfRh5 antibody, also pulled down PfRipr (Fig. 2E, left panel) but not PfRh2a or PfRh2b, two other members of PfRh protein family (Fig. 2E, right panel). Using culture supernatants, a similar immunoprecipitation experiment with the anti-HA antibodies to pull down PfRiprHA also co-precipitated the 45 kDa doublet of PfRh5 but not PfRh2a or PfRh2b (Fig. 2F). The reciprocal experiment using culture supernatants in which anti-PfRh5 antibody was used for immunoprecipitation detected HA-tagged PfRipr but not PfRh2a or PfRh2b in the pull-down sample (Fig. 2G). As an additional control, immunoprecipitaion of the culture supernatant with rabbit anti-PfRh2a/b antibody recognizing the 85 kDa fragment of both PfRh2a and PfRh2b was performed [24]. The anti-PfRh2a/b antibodies immunoprecipitated the 85 kDa PfRh2a/b but did not pull-down PfRh5 or PfRipr, further confirming that the PfRh5/PfRipr complex was specific. It was not possible to determine with these experiments whether the PfRipr/PfRh5 proteins associate in schizont stage parasites or at a later stage. However, since PfRh5 and PfRipr appear to be located in different compartments within the schizont (see below section), it is likely that they form a complex during detergent extraction as has been shown for the AMA1 and RON complex [43], [44], [45].

### PfRipr is a cysteine-rich protein that contains epidermal growth factor-like domains

**Figure 3:**
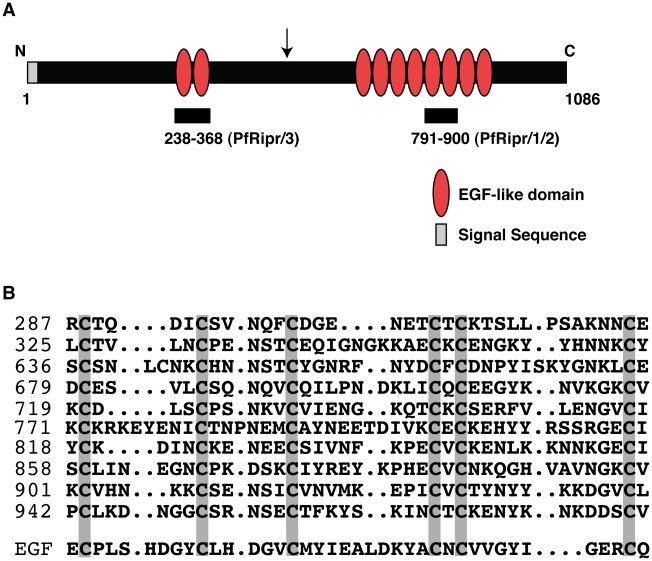
PfRipr protein has ten EGF-like domains. (A) Diagram shows the characteristics of PfRipr and the region used to generate the recombinant proteins. The arrow corresponds to the approximate position of the PfRipr cleavage site. (B) Alignment of the ten EGF-like domains within PfRipr.

PfRipr is highly conserved in *Plasmodium spp.* and the gene is syntenic with other genes, all of which are annotated as hypothetical (http://plasmodb.org). The full-length PfRipr protein consists of 1,086 amino acids with a molecular weight of 126 kDa. It has a putative hydrophobic signal sequence at the N-terminus consistent with it being secreted and the rest of the protein contains 87 cysteine-residues, many of which are clustered in epidermal growth factor (EGF)-like domains (Fig. 3A and B). There are ten EGF-like domains in the protein with two in the N-terminal region and eight clustered towards the C-terminus (Fig. 3A). An EGF domain has 6 cysteine residues and the position of each is relatively conserved in the ten EGF-like domains of PfRipr (Fig. 3B) [46], [47]. Processing of PfRipr occurs in the area between the second and third EGF-like domain (Fig. 3A); however, the processed N- and C-terminal regions remain associated as peptides from both regions were obtained in the mass spectrometry analysis of the immuno-precipitated PfRh5/PfRipr complex (Table 1) and the two regions were co-immunoprecipitated (see below).

### The N- and C-terminal domains of PfRipr form a complex

**Figure 4:**
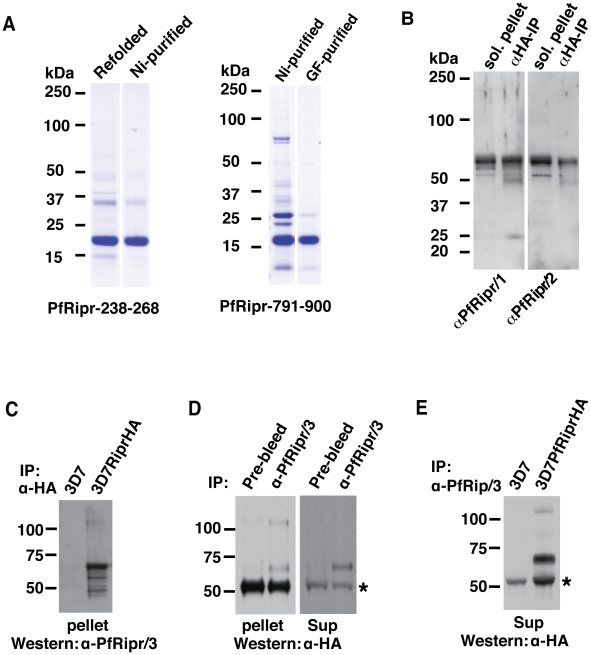
The N-terminal and C-terminal cleaved polypeptides remain associated after cleavage. (A) Amino acids 238-368 and 791-900 of PfRipr that encompassed the N-terminal first two EGF-like domains and C-terminal two EGF-like domains respectively were expressed as recombinant proteins in *E. coli* and purified for immunizing rabbits. The proteins were stained with Coomassie blue. (B) Rabbit polyclonal antibodies (anti-PfRipr/1 and anti-PfRipr/2) raised against the recombinant protein recognize native PfRipr. (C) Rat anti-HA antibody affinity beads were used to specifically immuno-precipitate proteins solubilized from schizont stages parasites of 3D7 and 3D7RiprHA lines and the bound materials immunoblotted and probed with rabbit anti-PfRipr/3 antibodies. (D) Rabbit anti-PfRipr/3 antibodies, in conjunction with protein G Sepharose, was used to specifically immuno-precipitate the proteins solubilized from schizont stages of 3D7 (panel 1) or culture supernatants (panel 2) and then the bound material was immunoblotted and probed with mouse anti-HA antibodies. The IgG from the pre-bleed serum of the same rabbit was used as a control. (E) Rabbit anti-PfRipr/3 antibodies, in conjunction with protein G Sepharose, was used to specifically immuno-precipitate the proteins from culture supernatant of 3D7 and 3D7RiprHA parasites and the bound material immunoblotted and probed with mouse anti-HA antibodies. The protein marked with a * in the panel D and E is protein G eluted from the beads that cross-reacts with the anti-HA antibodies.

To provide additional tools to probe the function of PfRh5/PfRipr complex, we expressed amino acids 238–368 of PfRipr that encompass the two EGF-like domains in the N-terminus as well as amino acids 791–900 of PfRir that encompass two EGF-like domains in the C-terminus (Fig. 3A), in *E. coli* as a recombinant protein tagged at the N-terminus with six histidines. The recombinant proteins of approximately 17 kDa were purified (Fig. 4A) and used to immunise rabbits. The anti-PfRipr/1 and -PfRipr/2 IgG antibodies, raised in two rabbits, were tested by western blot against solubilised pellets from schizont stages and a protein band of approximately 65 kDa was observed (Fig. 4B). Specificity of the antibodies was confirmed with HA-tagged PfRipr immunoprecipitated from culture supernatant with anti-HA antibodies and in both cases a 65 kDa protein was detected (Fig. 4B). This was the molecular weight expected for the processed C-terminal region of PfRipr; however, the mature full-length protein of 123 kDa was not easily observed as it was mostly processed at late schizont stage and in the supernatant. The anti-PfRipr/3 antibody has low reactivity to the denature protein but recognizes native PfRipr well.

The anti-PfRipr/3 was therefore used in co-immunoprecipitation experiments to confirm that the cleaved N-terminus and C-terminus of PfRipr remain associated. Immunoprecipitation of the HA-tagged C-terminus of PfRipr from 3D7RiprHA parasites using anti-HA antibodies co-precipitated a fragment of approximately 60 kDa recognized by the anti-PfRipr/3 (Fig. 4C). The size of the fragment is expected for the N-terminal domain of the processed PfRipr. The full-length mature protein of approximately 125 kDa was also observed as expected since it should react with both the anti-PfRipr/3 and anti-HA antibodies. The reciprocal experiment to immunoprecipitate the N-terminal domain with schizont material using anti-PfRipr/3 co-precipitated the 65 kDa C-terminal fragment and the 125 kDa full length protein (Fig. 4D, left panel). An identical experiment using culture supernatant co-precipitated the 65 kDa C-terminal domain (Fig. 4D, right panel); the full-length protein was not detected in this case, presumably because it was processed. Immunoprecipitation using anti-PfRipr/3 and immunoblots with anti-HA antibodies detected the expected bands of the 65 kDa C-terminal domain and the 125 kDa full-length protein in 3D7RiprHA but not from the parental parasite line 3D7 (Fig. 4E), further confirming the specificity of the antibodies. Together these experiments show that PfRipr is processed into two fragments of approximately 65 kDa each and they remain associated with each other to form a complex with PfRh5, consistent with mass spectrometry results (Table 1). Their association does not involve intermolecular disulfide linkages (Fig. 2C).

**Figure 5:**
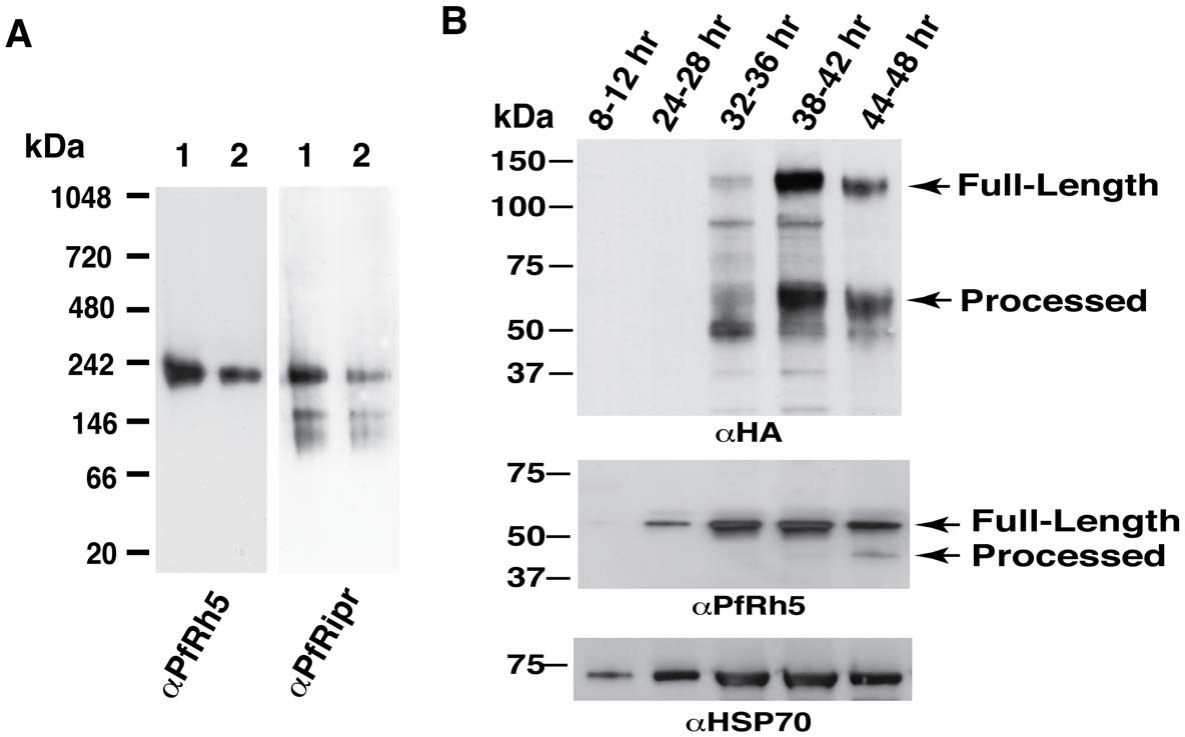
Protein expression patterns of PfRh5 and PfRipr are consistent with the complex formation. (A) Both anti-PfRh5 and anti-PfRipr antibodies recognize the same complex resolved by blue native gel electrophoresis. (B) Both PfRh5 and PfRipr are expressed late in the blood stage life cycle of parasite development. Synchronized PfRiprHA parasites were distributed into six 5 ml cultures. One was harvested immediately after synchronization (8 – 12 hr ring stage). The second was harvested 16 hr post synchronization, and the third 8 hr later. The rest of the time points were harvested every 6 hr until the end of growth cycle. Proteins were extracted from saponin pellets, separated by SDS-PAGE and transferred to nitrocellulose membrane. The membrane was probed with monoclonal anti-HA antibody (top panel) to detect PfRiprHA and then stripped and re-probed to detect PfRh5 (middle panel) and PfHsp70 (bottom panel). Both PfRh5 and PfRipr proteins are expressed primarily in late schizonts.

The anti-PfRipr/1 was used in western blot to probe the PfRipr separated on a blue native gel (Fig. 5A). Indeed anti-PfRipr/1 recognizes the same protein complex detected by anti-PfRh5, with the size of the complex (approximately 200 kDa) consistent with that seen in Fig. 1B.

Expression of PfRipr in the asexual blood stage life cycle was determined using synchronised ring stages parasites from 3D7RiprHA. Saponin pellets were prepared from the samples taken during the parasite life cycle as indicated (Fig. 5B). Anti-HA antibody detected both the 125 kDa full-length and 65 kDa fragment of PfRipr and expression was evident in late trophozoites to schizonts, the expected pattern for proteins that play a role in merozoite invasion [20]. The 65 kDa processed form appeared late in parasite blood stage development indicating that this processing event occurs in the schizont stage before merozoite egress. The same samples were probed for PfRh5, with which PfRipr forms a complex, and it showed a similar timing of expression but processing of this protein appears to occur late in schizogony, as observed earlier [20]. The similar pattern of PfRipr and PfRh5 expression late in the asexual blood stage life cycle of *P. falciparum* was consistent with formation of a complex between them either in the schizont stage or during merozoite egress before interaction of the parasite with the host cell.

### The PfRipr/PfRh5 complex is tightly associated with the membrane of *P. falciparum*

**Figure 6:**
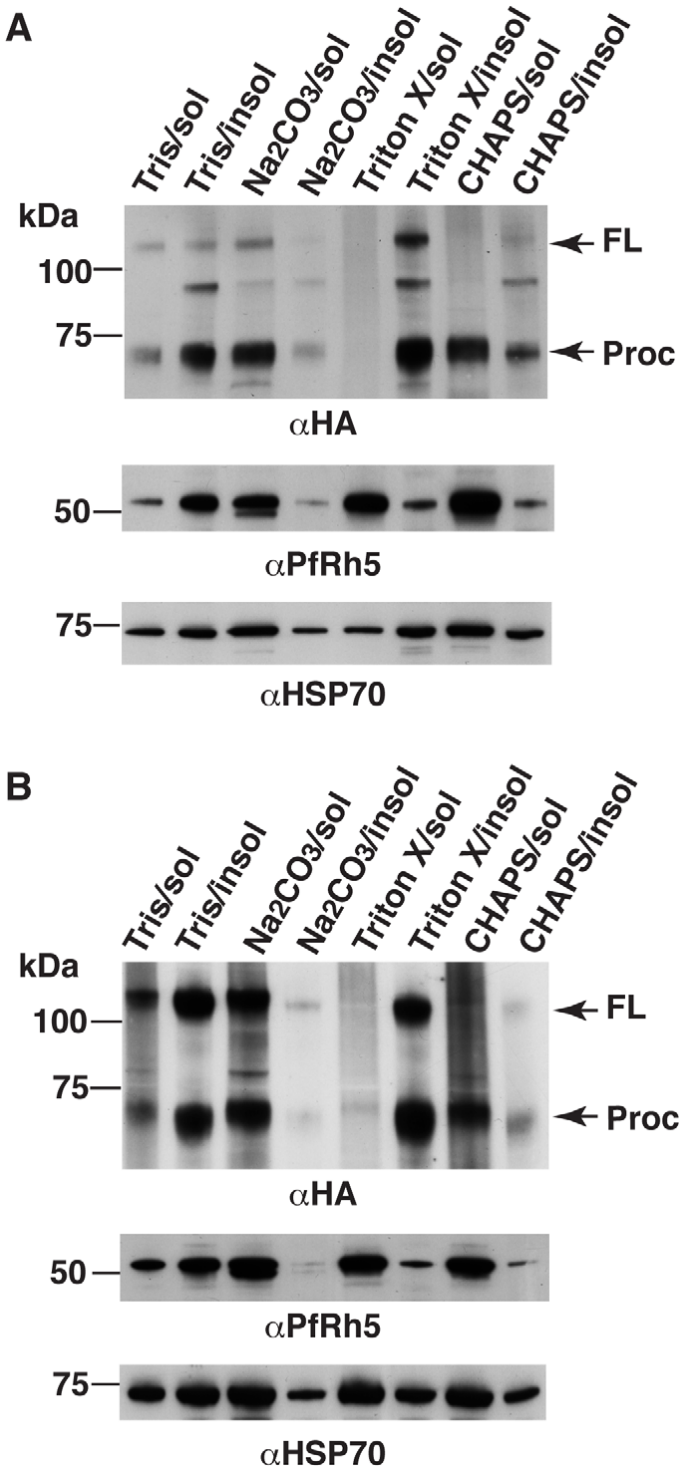
PfRipr and PfRh5 are peripherally associated with parasite membrane. (A) Differential solubilization of proteins from pellet prepared by hypotonic lysis of red blood cells that had been infected by the late schitzont stage 3D7-RiprHA parasite. (B) Differential solubilization of proteins from saponin pellets of late schitzont stage 3D7RiprHA parasites.

Whilst both PfRipr and PfRh5 have a signal sequence at the N-terminus for entry into the ER, neither has a hydrophobic domain that would provide the means for anchoring in the membrane. As PfRh5 binds to the host erythrocyte [18], [19], [20] it would be expected that the PfRh5/PfRipr complex would be bound to the membrane of the merozoite to provide a junction and perhaps transmit an appropriate signal [37]. To determine if this complex was associated with a membrane, we used differential solubilisation of the parasite to determine the properties of the PfRipr/PfRh5 complex (Fig. 6). *P. falciparum* parasites were hypotonically lysed in water and centrifuged to obtain a pellet and supernatant fraction. Treatment of the pellet with 10 mM Tris showed that both PfRipr and PfRh5 remained predominantly in the pellet fraction suggesting they were associated with a membrane (Fig. 6A). However, treatment of the pellet fraction with Na_2_CO_3_ showed that both proteins moved to the soluble fraction suggesting they were extrinsically associated as peripheral membrane proteins of the parasite. PfRipr was mainly insoluble in Triton X-100 but the smaller processed form of the protein was more soluble in CHAPs detergent. In contrast, PfRh5 was more soluble in Triton X-100 and CHAPS implying either PfRipr/PfRh5 complex involves hydrophobic interactions that can be disrupted by non-ionic detergents [48], or PfRipr and PfRh5 have different membrane association properties. Similar solubility characteristics were obtained from saponin treated parasites isolated with saponin treatment (Fig. 6B). In summary, these results showed that the PfRh5/PfRipr complex was peripherally associated with the parasite membrane and it is possible that another protein(s) may be involved in the membrane attachment of the PfRh5/PfRipr complex.

### PfRipr is located in the apical organelles of the merozoite

**Figure 7:**
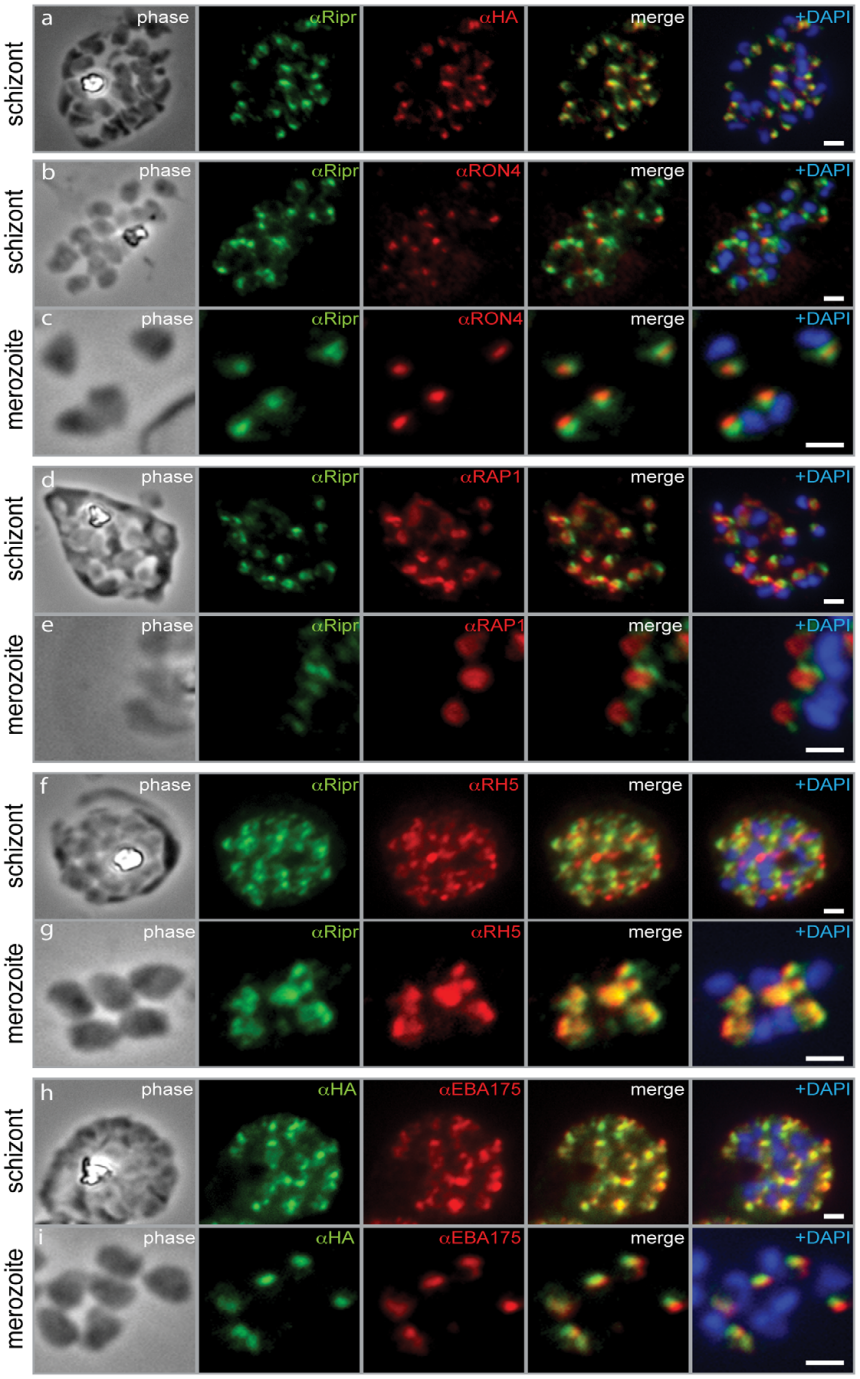
PfRipr localizes to the apical end of merozoites. a) Rabbit polyclonal anti-PfRip antibody (anti-PfRipr/1) recognizes HA-tagged PfRiprHA. b) PfRipr does not co-localize with the rhoptry neck protein RON4 in schizonts. c) PfRipr does not co-localize with the rhoptry neck protein RON4 in merozoites. d) PfRipr does not co-localize with the rhoptry bulb protein, RAP1 in schizonts. e) PfRipr does not co-localize with the rhoptry bulb protein, RAP1 in merozoites. f) PfRipr partially co-localizes with PfRh5 in the schizonts. g) PfRipr mainly co-localizes with PfRh5 in purified merozoites. h) PfRipr co-localizes with the micronemal marker, EBA175, in schizonts. i) PfRipr does not co-localize with the micronemal marker, EBA175, in merozoites.

With anti-PfRipr antibodies, we performed immuno-fluorescence assays to determine the subcellular localization of PfRipr with respect to known markers of compartments such as micronemes and rhoptries, organelles that are important for erythrocyte invasion. We firstly confirmed that the HA-tagged PfRipr protein showed the same localisation when detected using both anti-HA and anti-PfRipr/1 antibodies, with both showing a punctate pattern in schizonts (Fig. 7, panel a). Co-labelling of schizonts and merozoites with PfRipr and the rhoptry neck marker RON4 (Fig. 7, panel b and c) or the rhoptry body marker, RAP1 (Fig. 7, panel d and e), both showed close alignment but no significant overlap of signal. These results suggest that PfRipr localises to the apical tip of the merozoite in an area distinct from the very apical tip and the bulb of the rhoptries. Comparison of the subcellular localisation of PfRh5 and PfRipr in schizont stages showed that whilst there appeared to be some overlap, there were substantial areas of the punctate pattern that were clearly in a separate part of the cell (Fig. 7, panel f). In contrast, PfRh5 and PfRipr showed substantial co-localisation in merozoites suggesting that the two proteins may form a complex in late schitzonts or merozoits (Fig. 7, panel g). Furthermore, the substantial overlap of PfRipr and the micronemal protein EBA-175 in the schizont stage suggest that PfRipr may be predominately present within this organelle at this stage (Fig. 7, panel h). However, in merozoites they clearly did not co-localise although there was some overlap (Fig. 7, panel i). Taken together, these data are consistent with PfRipr localising to micronemes and being released in merozoites.

**Figure 8:**
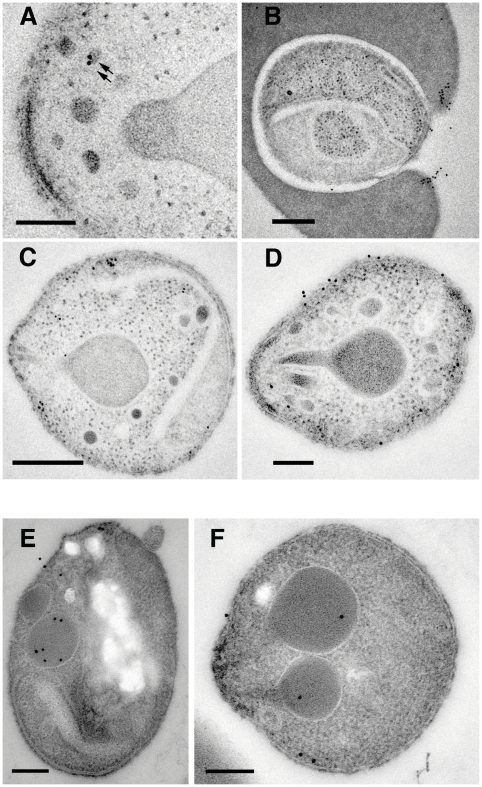
Subcellular localization of PfRipr and PfRh5 by immuno-electron microscopy. (A) Localisation of HA-tagged PfRiprHA using anti-HA antibodies. The protein localizes to electron dense structures at the apical end of the merozoite that resemble micronemes (arrows). Mn, micronemes: Rh, rhoptries. (B) PfRiprHA can be detected at the leading edge of the tight junction formed between the erythrocyte and merozoite in a merozoite in the process of invading a red blood cell. Nu, nucleus: TJ, tight junction. (C) PfRiprHA can be observed concentrating in structures at the apical end at the periphery of the parasite. Rh, rhoptries. (D) Some merozoites show labeling of PfRipHA on the merozoite surface. (E) and (F) Localisation of PfRh5 using monoclonal anti-PfRh5 antibody. PfRh5 localises to rhoptries but was also observed at the apical surface, suggesting it was being released in free merozoites. Rh, Rhoptries. Scale bar is 200 nm.

To more finely determine the subcellular localisation of PfRipr, immuno-electron microscopy was performed on 3D7RiprHA merozoites (Fig 8A to D and Fig. S2). HA-tagged PfRiprHA was observed towards the apical end of merozoites in more electron dense structures, suggesting localisation in micronemes (Fig. 8A). In merozoites fixed during erythrocyte invasion we observed a concentration of PfRipr reactivity at the leading edge of the tight junction, consistent with the protein being shed and released into the culture supernatant during invasion (Fig. 8B). This suggests that PfRipr was shed from the merozoite surface as the tight junction moves across its surface so that it concentrates at the posterior end before sealing of the parasitophorous vacuole membrane. Whilst there were clearly concentrations of PfRipr in structures at the apical end (Fig. 8C) we also observed considerable surface localisation on free merozoites suggesting it is released prior to invasion (Fig. 8D). In comparison PfRh5 seems to localise to rhoptries (Fig. 8E, F) as we have observed previously [20]; however, there was also some labelling at the apical surface, suggesting this protein was also being released prior to invasion (Fig. 8 E, F). These results are consistent with that observed by immuno-fluorescent microscopy (Fig. 7). From these localization data, we suggest that PfRipr and PfRh5 localise to separate subcellular compartments and thus do not form a complex in schizont stage parasites. Presumably following fusion, or secretion, of these distinct compartments, they then come together in merozoite or late schizont stages. Once complexed and at the apical end of the merozoite, they interact with a receptor on the erythrocyte surface. Such an interaction between proteins originating in separate organelles is not unprecedented, with one prominent example being the interaction between AMA1 and RON4 to form the invasion tight junction across apicomplexan species [43], [44], [45].

### Antibodies to PfRipr inhibit attachment and invasion of *P. falciparum* merozoites

**Figure 9:**
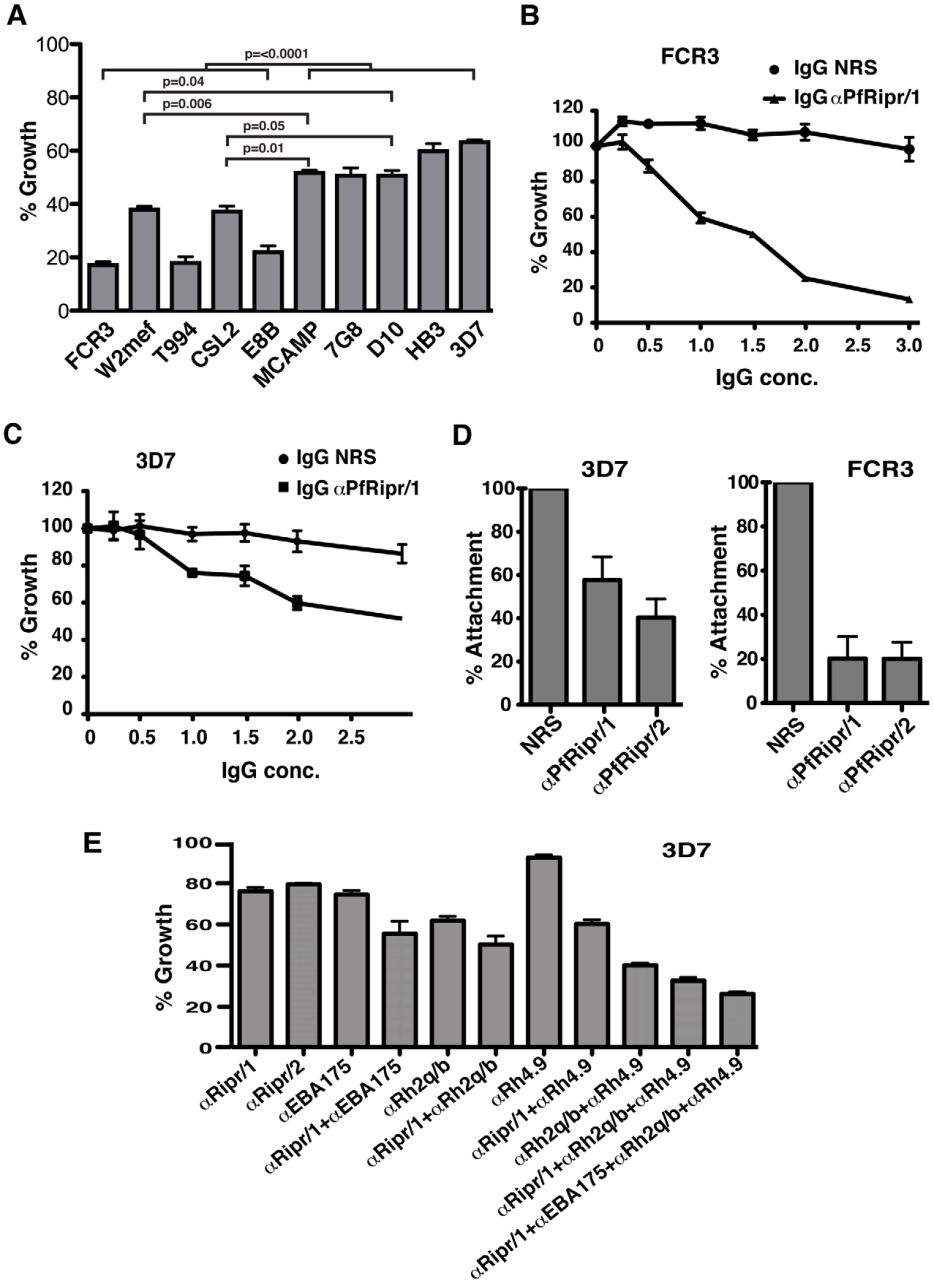
Antibodies to PfRipr inhibit attachment of merozoites to erythrocytes and parasite growth. (A) Anti-PfRipr/1 antibodies inhibit invasion of *P. falciparum* strains into erythrocytes. Shown are growth inhibition assays of the parasite strains FCR3, W2mef, T994, CSL2, E8B, MCAMP, 7G8, D10, HB3 and 3D7. The final antibody concentration is 2 mg/ml. (B) Titration of anti-PfRipr/1 antibodies in growth inhibition assays of the FCR3 strain. (C) Titration of anti-PfRipr/1 antibodies in growth inhibition assays of the 3D7 strain. (D) Pre-incubation of purified merozoites from 3D7 strain (left panel) and FCR3 strain (right panel) with protein-A purified antibodies raised against recombinant PfRipr inhibited merozoite attachment to red blood cells. Protein-A purified antibodies from normal serum were used as a negative control. The final antibody concentration is 2 mg/ml. (E) Various combinations of anti-PfRipr/1, EBA-175, PfRh2a/b and PfRh4 antibodies increase inhibitory activity for the 3D7 strain of *P. falciparum*. The final concentration of each antibody is 1 mg/ml. In all the cases, each graph represents three independent experiments done in triplicate with each normalised to the negative control (Protein A purified IgG from normal rabbit serum). The error bars represent standard error of the mean of the three independent experiments.

To determine if PfRipr was required for development of *P. falciparum*, we tested the ability of anti-PfRipr/1 and/2 antibodies (Fig. 3) to block parasite growth (growth inhibition assays, GIA [7]) using the *P. falciparum* strains FCR3, W2mef, T994, CSL2, E8B, MCAMP, 7G8, D10, HB3, and 3D7 (Fig. 9A). The antibodies inhibited parasite growth of all strains tested and significantly, FCR3 was inhibited to 80% whilst in comparison 3D7 was inhibited to 35% with αPfRipr/1 at 2 mg/ml (Fig. 9A). The inhibition observed for 3D7 was comparable to that observed for other antibodies raised to regions of the PfRh or EBL protein families [7], [12]. Similar results were observed for 3D7 using the αPfRipr/2 (data not shown). As for the anti-PfRipr/1 and/2 antibodies, rabbit polyclonal to the N-terminal EGF-like domains (anti-PfRipr/3) also inhibited parasite growth for all strains tested (Fig. S3). The αPfRipr/1 antibody was titrated in GIAs in comparison with IgG from normal serum for both FCR3 and 3D7 parasite strains (Fig. 9B and C). Growth of FCR3, a parasite that invades preferentially by sialic acid-dependent pathways, was almost completely abolished at 3 mg/ml and significant inhibition still remained at 1 mg/ml (40%) (Fig. 9B). In comparison, the 3D7 parasite strain, which can efficiently use sialic acid-independent invasion pathways primarily by using the ligand PfRh4 and complement receptor 1 [49], was inhibited at significantly lower levels of 50% at 3 mg/ml and this decreased to 20% at 1 mg/ml of antibody (Fig. 9C). This suggests that the PfRipr/PfRh5 complex may be more functionally important in *P. falciparum* strains that efficiently use sialic acid-dependent invasion pathways, which is supported by our finding (Fig. S4) that anti-PfRipr/1 inhibits W2mef, a sialic acid-dependent strain, more effectively than W2mefΔ175, a sialic acid-independent strain [8]. Among other *P. falciparum* strains tested, the αPfRipr/1 antibody also exhibited significantly higher inhibitory activity for those that invade erythrocytes preferentially using sialic acid-dependent receptors (ie. glycophorins), which includes T994, CSL2 and E8B (Fig. 9A) [9].

To investigate the mechanism of inhibition by anti-PfRipr antibodies, we performed merozoite attachment assays (Fig. 9D). Viable merozoites from 3D7 and FCR3 parasites were purified [45], [50] and mixed with erythrocytes in the presence of αPfRipr/1 or αPfRipr/2 antibodies. Both antibodies inhibited merozoite attachment for 3D7 to a significant level (45% and 60% respectively) (Fig. 9D, first panel). In contrast, the same antibodies inhibited attachment of FCR3 merozoites to a considerably greater extent (80% for both antibodies) (Fig. 9D, second panel). The level of inhibitory activity observed with the αPfRipr/1 and αPfRipr/2 antibodies for 3D7 and FCR3 parasites was similar to that observed in the growth inhibition assays (Fig. 9A, B and C) demonstrating that inhibition was occurring at merozoite invasion rather than during growth of the parasite. This inhibition was likely due to interference of these antibodies with the function of the PfRipr/PfRh5 complex since we could not detect PfRipr binding to erythrocytes while consistently observing PfRh5 binding (data not shown) [20]. It was possible that the PfRipr/PfRh5 complex disassociated on binding of PfRh5 to red blood cells. To test this possibility we purified the PfRh5/PfRipr complex using ion-exchange chromatography (Fig. S5A) and tested its binding to red blood cells (Fig. S5B). This showed that PfRh5 bound to red blood cells but PfRipr was not detected suggesting that the complex was disrupted on binding.

The region of PfRipr to which the αPfRipr/1, 2 antibodies were raised was from the 3D7 strain of *P. falciparum*; however, this domain does not show any polymorphisms compared to other strains so far sequenced (http://plasmodb.org/and http://www.broadinstitute.org/) (Fig. S6). Also we did not observe any cross-reactivity of the antibodies with other proteins that contain EGF-like domains such as MSP1 (data not shown). This was not surprising as the only conserved amino acids are the six cysteine residues that define each EGF-like domain (Fig. 3). Therefore the differences in inhibition observed in GIA with the various strains was unlikely to be due to cross reactivity with other proteins containing EGF-like domains or polymorphisms within this region of PfRipr. It more likely reflects the reliance of these strains on the PfRh5/PfRipr complex to mediate a specific invasion pathway in comparison to the function of other members of the PfRh and EBA protein families [7], [51]. To test this we used a combination of antibodies raised to PfRipr, EBA-175 [51], PfRh4 [13], [49], PfRh2a and PfRh2b [7] to determine if they increased the level of inhibition in GIAs for 3D7 parasites (Fig. 9E). Both αPfRipr/1 and αPfRipr/2 antibodies at 1 mg/ml inhibited 3D7 parasite growth to 24 and 21% respectively (Fig. 9E), similar to our previous experiments (Fig. 9C). Anti-EBA-175 antibodies on its own also inhibited 3D7 parasite growth to 25%. Anti-PfRh2a/b and PfRh4 antibodies inhibited the 3D7 parasite growth at 39% and 8% respectively. The combination of αPfRipr/1 with αEBA-175 antibodies showed an additive inhibition of 45%. This was a similar result to that observed for the combination of αPfRipr/1 with αPfRh2a/b or αPfRh4 antibodies (50% and 40% respectively). Significantly, a combination of αPfRipr/1, αPfRh2a/b and αPfRh4 as well as αPfRipr/1, αEBA-175, αPfRh2a/b and αPfRh4 showed a much higher level of inhibition (67% and 74% respectively). This additive effect was consistent with parasites using multiple invasion pathways to gain entry to the erythrocyte. A similar additive inhibitory effect was obtained with FCR3 parasites (Fig. S7).

Our inability to disrupt the *PfRipr* gene (Fig. S8 and S9) suggests the function of PfRipr and thus the PfRipr/PfRh5 complex is essential for parasite invasion and survival. Therefore PfRipr represents a novel protein that plays a critical role in merozoite invasion and thus it is a new candidate for a combination vaccine with other PfRh and EBL proteins to produce antibody responses to block a broad array of invasion pathways.

## Discussion

Invasion of *P. falciparum* into host erythrocytes requires specific ligand-receptor interactions that identify the appropriate host cell followed by activation of the parasite actomyosin motor for entry (see for review [2]). The PfRh family of proteins are important ligands that bind directly to specific receptors on the red blood cell during invasion [4], [5], [7], [9], [11], [13], [16], [18], [19], [20], [22], [49]. Differential expression and activation of these ligands provide a mechanism of phenotypic variation to evade host immune responses and to circumvent the polymorphic nature of the red blood cell surface in the human population [7], [8], [39]. PfRh5 is an important member of the PfRh family that appears to be essential in *P. falciparum* [19], [20]. This ligand binds to an unknown receptor and plays a key role during merozoite invasion; however, in contrast to all other PfRh family members, it lacks an identifiable transmembrane region, suggesting that it may function in a complex with another protein(s) [20]. We have shown here that PfRh5 exists in a membrane-associated complex with a novel cysteine-rich protein that we have called PfRipr.

The ability of PfRipr antibodies to inhibit merozoite attachment and invasion and our inability to genetically disrupt the corresponding gene implies that this protein plays an important role in these processes. Before invasion, the merozoite undergoes a step of irreversible attachment to the host red blood cell and current evidence suggests that the PfRh and EBL protein families play a key role in this process [37], [45]. Attachment triggers downstream events presumably through signalling from the cytoplasmic domain of each molecule [37], [45]. Merozoite attachment can be inhibited with anti-PfRipr antibodies consistent with the PfRh5/PfRipr complex playing a role in the irreversible binding to erythrocytes and commitment to invasion. The ability to inhibit merozoite invasion was not due to cross reactivity of the antibodies with other proteins such as MSP-1, that also have EGF-like domains. Whilst neither PfRh5 nor PfRipr has a transmembrane region they are tightly associated with the membrane, suggesting they most likely complex with another protein(s) that would hold the complex on the parasite surface after their release from the apical organelles. This would link the parasite to the erythrocyte by binding of PfRh5 to its specific receptor during merozoite invasion.

The subcellular localisation of PfRh5 and PfRipr appears to be different in schizont stages of the parasite suggesting that they may not form a complex until egress of the merozoite and activation of erythrocyte invasion. PfRh5 was present within the rhoptries whilst PfRipr appears to be in micronemes at the apical end and these proteins would be released during apical interaction where they could associate and form a complex. This is similar to the formation of the AMA-1 and RON complex in both *T. gondii* and *P. falciparum* where the former protein is located in micronemes and the latter in the neck of the rhoptries [43], [44], [52], [53]. Components of the RON complex translocate onto and under the erythrocyte membrane forming a bridge across the host cell to the invading merozoite through AMA-1 and this is associated with the tight junction. It is possible that PfRh5/PfRipr interacts with a membrane associated protein and our immuno-electron microscopy analysis has suggested that the complex is at the leading edge of the tight junction (i.e. basal to the merozoite) and shed into the culture supernatant, or onto the exterior of the red blood cell, before membrane fusion to form the enclosing parasitophorous vacuole membrane at the posterior end of the invading merozoite.

The PfRh and EBL proteins may have overlapping functions as loss of EBA-175 can be compensated by increased function of PfRh4 [8], [51], [54]. Furthermore, different parasite lines can differentially express these proteins and in some cases polymorphisms affect their function or potentially alter receptor selectivity [7], [8], [19], [28], [38]. For example, both PfRh2a and PfRh2b are not expressed in FCR3 line [7] and additionally, both EBA140 and EBA-181 encode polymorphisms that greatly reduce their binding to the red blood cell surface and render them non-functional when tested in growth inhibition assays with specific antibodies [55]. Similarly, in the W2mef parasite line PfRh4 is not expressed and polymorphisms of EBA-140 and EBA-181 also weaken their binding abilities and function in invasion [55]. Since both FCR3 and W2mef have a reduced complement of functional PfRh and EBL proteins, one would predict that these parasites would have greater reliance on the PfRh5/PfRipr complex for their invasion. That is what we have observed in GIAs with the anti-PfRipr antibodies that efficiently inhibit merozoite invasion of these strains. In addition, the additive effect observed with anti-PfRipr antibody in combination with antibodies raised against other PfRh and/or EBA in GIAs would be consistent with the PfRh5/PfRip complex having a similar function to other members of the PfRh and EBL protein families [54]. However, in contrast to the other PfRh and EBL proteins, both PfRh5 and PfRipr could not be genetically disrupted, pointing to the possibility that the complex may also play a broader role that is essential to the parasites.

The PfRh1, PfRh2a and PfRh2b proteins undergo a complex series of proteolytic cleavage events in the schizont when trafficked to the neck of the rhoptries as well as during merozoite invasion and these events are important in their function [9], [12], [24]. In the case of PfRh2a and PfRh2b, the proteins are processed to produce the N-terminal binding domain that remains associated with the C-terminal domain [24]. It makes sense for N-terminal binding domain and C-terminal domain with a transmembrane region to associate together after processing for the parasites to attach to the erythrocytes. PfRh5 is also located in the neck of the merozoite rhoptries and is processed to a 45 kDa binding region [20]. Here we showed that the 45 kDa binding region forms a complex with PfRipr. Since neither of them contain a transmembrane region, it is likely that the complex binds another protein(s) to link it to the merozoite surface for PfRh5 to bind erythrocyte during invasion. Our results that the PfRipr/PfRh5 complex is peripherally attached to the parasite membrane are consistent with this possibility.

Epidermal growth factor-like domains are found widely throughout eukaryotes and are defined by six cysteine residues that link to form a tightly folded structure [46], [47]. In *Plasmodium spp.* this domain is contained in a large number of proteins in different stages of the parasite lifecycle. The merozoite surface antigen 1 (MSP-1) has two EGF-like domains and it is localised to the merozoite surface where antibodies can access it and inhibit invasion, which has suggested it is involved in interacting with the erythrocyte surface [56], [57]. Other proteins with EGF-like domains include the P28 family that protect the parasite from the harsh proteolytic environment in the mosquito gut [58]. PfRipr would not play such a protective role as it is released only during invasion. Instead, it may act as a scaffold on which PfRh5 can be mounted so that it is displayed for binding to its erythrocyte receptor. As the N-terminal and C-terminal of PfRipr remains associated after processing, it is likely that the EGF-like domains are responsible for their association as well as interaction with PfRh5. PfRipr has previously been predicted to act as a parasite adhesin by a bioinformatic method based on protein physiochemical properties [59]. However, we could not detect direct binding of PfRipr to erythrocytes and our results suggest that the PfRipr/PfRh5 complex dissociates upon PfRh5 binding to red blood cells. This may be similar to the processed N-terminus of PfRh2a and PfRh2b that form a complex with the C-terminus of the same proteins. However, only the free processed N-terminus can be detected in erythrocyte binding assays suggesting that this complex also disassociates [24].

The PfRh and EBL protein families function in binding the erythrocyte and activating the invasion process [54], [55]. It has been proposed that a combination of these ligands may be a useful blood stage vaccine against *P. falciparum* malaria. Indeed it has been shown in immunisation studies that vaccination with combined portions of EBA-175, PfRh2a/b and PfRh4 proteins can raise antibodies that efficiently block merozoite invasion [54]. The functional importance of PfRh5 has also led to suggestion that it has potential to be included in such a combination vaccine. However, this has not been tested due to the difficulty of producing functional protein. Identification of the PfRh5/PfRipr complex and the ability of anti-PfRipr antibodies to block merozoite invasion open the possibility for its inclusion in a combination vaccine. Indeed, the efficient inhibition of invasion was observed when a combination of antibodies to PfRipr, PfRh2a/b and PfRh4 was used. This further supports PfRipr as a new vaccine target, especially in a combination approach to efficiently block merozoite invasion.

## Materials and Methods

### Ethics statement

Antibodies were raised in mice and rabbits under the guidelines of the National Health and Medical Research Committee and the PHS Policy on Humane Care and Use of Laboratory Animals. The specific details of our protocol were approved by the Royal Melbourne Hospital Animal Welfare Committee.

### Parasite culture

*P. falciparum* asexual parasites were maintained in human erythrocytes (blood group O+) at a hematocrit of 4% with 10%(w/v) AlbumaxTM (Invitrogen) [60]. 3D7 is a cloned line derived from NF54 from David Walliker at Edinburgh University. FCR3 is a cloned line. Cultures were synchronised as previously described [61]. Culture supernatants enriched in parasite proteins were prepared by growing synchronized parasite cultures to high parasitemia, typically to 5% and allowing schizonts to rupture. Harvested culture supernatant was spun at 1200 rpm to remove residual erythrocytes and then 10,000 rpm to remove insoluble materials before use for experiments. Total proteins from schizont stage parasites were obtained by saponin lysis of infected erythrocytes.

To prepare culture supernatants for affinity purification of the PfRh5 complex, the PfRh5HA parasite line was synchronized, grown to late schizont stage in normal culture conditions and then culture medium was replaced by RPMI without Albumax to allow the schizonts to rupture [5]. This culture supernatant has a minimal amount of BSA to facilitate purification of the PfRh5 complex.

### Purification and identification of PfRh5 complex

Culture supernatant (900 ml), prepared as described above, was dialysed against 12.5 mM Tris.Cl pH 7.2 overnight at 4°C and loaded onto a 15 ml Q-Sepharose column (GE Heathcare) equilibrated with 12.5 mM Tris.Cl, pH 7.2. The bound proteins were eluted with NaCl in 12.5 mM Tris.Cl pH 7.2. PfRh5HA was eluted with 350 mM NaCl. The eluted PfRh5HA-containing fractions were further purified using anti-HA affinity matrix (Roche Apply Science) and the bound proteins eluted with 0.1 M glycine, pH 2.6. The eluted proteins were then subjected to trypsin digestion and analyzed by mass spectrometry (LC-MS/MS) and proteins identified by database searches [42].

### Gel filtration chromatography and blue native gel electrophoresis

Gel-filtration chromatography was performed on an analytical Superdex 200 column (24 ml, GE Healthcare) and proteins were eluted with PBS or Tris buffer. Blue Native Gel Electrophoresis was conducted using the company's protocols (Invitrogen). NativePAGE^TM^ Novex 4–16% or 3–12% Bis-Tris gels were used to resolve the proteins and the NativeMark^TM^ Unstained Protein Standard used as molecular weight markers.

### Generation of *P. falciparum* expressing HA-tagged PfRipr

To attach a triple HA tag (3xHA) to the 3′ end of the *Pfripr* gene, an 844 bp fragment of *Pfripr* was amplified from 3D7 genomic DNA using the primers 5′-ATCCCGCGGTGAATGTATATTAAATGATTATTG-3′ and 5′-TTATCTCGAGATTCTGATTACTATAATAAAATACATTTTC-3′ (*Sac* II and *Xho I* restriction sites underlined). The DNA fragment was digested with *Sac* II and *Xho* I, and cloned into pHAST, a derivative of pGEM-3Z containing a 3xHA tag and single Strep II tag in tandem. Parasites were transfected as described previously [62], [63]. Successful integration of the 3xHA tag was determined by Southern and Western blot analysis using a mouse monoclonal anti-HA antibody.

### Immunoprecipitation and immunoblotting

Immunoprecipitation of PfRh5 from culture supernatant was performed using anti-PfRh5 monoclonal antibody (clone 2F1) coupled to Minileak resin (KEM-En-Tec). Briefly, 1.5 ml culture supernatants from both 3D7 and 3D7-PfRiprHA parasite lines were incubated with 20 µl anti-PfRh5-Minileak resins at 4°C for 4 hr. Also 1.5 ml culture supernatant of 3D7-PfRiprHA parasites was incubated with just 20 µl Mini-bead as an additional control. After incubation, the samples were spun to remove the supernatant and the resin washed three times with PBS containing 0.1% Tween-20. Bound proteins were eluted with SDS sample buffer and separated by SDS-PAGE, transferred to nitrocellulose membrane and probed with a monoclonal anti-HA antibody (12CA5). The membrane was then stripped and re-probed with rabbit anti-Rh2a/b polyclonal antibodies recognizing 85 kDa domain. Immunoprecipitation of HA-tagged PfRipr from culture supernatant of 3D7-PfRiprHA was performed using rat anti-HA affinity matrix (Roche Applied Science). The culture supernatants from 3D7 parasites were used as a control. The bound material was analysed by western blot to probe for PfRh5 using monoclonal anti-PfRh5 antibody (2F1) or to probe for PfRipr using polyclonal antibodies raised against N-terminal fragment (α-PfRip/3). The material was also probed with anti-Rh2a/b polyclonal antibodies as a control. Immunoprecipitation of PfRipr from culture supernatant of 3D7-PfRipHA with α-PfRip/3 was performed using protein G. The IgG from a pre-bleed of the same rabbit was used as a control. Immunoprecipitation of PfRipr from solubilised saponin pellets of the 3D7RiprHA parasite line was performed similarly. The proteins were extracted from the saponin pellet using 2% n-Dodecyl-N, N-Dimethylamine-N-Oxide in PBS. Immunoprecipitation of culture supernatant from 3D7-PfRipHA with rabbit anti-Rh2a/b polyclonal antibodies was also performed with protein G. The bound material was probed for PfRh2a, PfRh2b, PfRh5 and PfRipr using corresponding antibodies raised in mice. The proteins were detected by enhanced chemiluminescence (ECL, Amersham Biosciences).

### Analyses of protein expression

Synchronized 3D7RiprHA early ring stage parasites were harvested and the red blood cells lysed using saponin. A second aliquot was harvested 16 hr later, the third aliquot another 8 hr later and subsequent samples every 6 hr later until the end of schizogony. Proteins were extracted from the saponin pellets using SDS-PAGE sample buffer, separated by 4-12% SDS-PAGE gels (Novagen) and transferred to a nitrocellulose membrane. The membrane was firstly probed with monoclonal anti-HA antibody for PfRiprHA and then stripped to probe for PfRh5 and PfHsp70 [64].

### Generation of recombinant PfRipr and antibody production

To produce recombinant C-terminal fragment of PfRipr comprise of amino acid 791-900, a 345 bp DNA fragment was amplified from genomic DNA prepared from 3D7 parasites using oligonucleotides (5′ CGCTAGCCATATGAATGAAGAAACAGATATTGTAAAATG 3′ and 5′ CGAGGATCCCTAATCTTCTAAAACACATTTTCC 3′). The resulting PCR fragment was cloned into pET14b vector (Novagen) with *Nde* I and *Bam* HI, transformed into BL21 RIL *E. coli* strain to express the recombinant PfRipr-791-900 as a hexa-His-tagged protein (Fig. 3 A). The His-tagged protein was purified from soluble lysate of bacteria cells by affinity purification on Ni-NTA agarose resin (Qiagen) followed by gel-filtration chromatography on Superdex^TM^ 75 column. To produce recombinant N-terminal fragment of PfRipr consisting of amino acid 238–368, a codon-optimised gene was synthesized and cloned into pET28a vector (Novagen) with *Nhe* I and *Bam*HI sites, transformed into BL21 RIL *E. coli* strain for expressing recombinant PfRip-238-368 as a hexa-His-tagged protein. The His-tagged protein was expressed in *E. coli* as inclusion body. Protein isolated from inclusion bodies was refolded *in vitro* and purified on Ni-NTA agarose resin (Qiagen) under native conditions. Both purified PfRip-791-900 and PfRip-238-368 proteins were used to immunise a rabbit. Rabbit immunoglobulins were purified on Protein A or G-Sepharose and buffer exchanged to PBS for subsequent experiments. The antibody production was done by the WEHI antibody production facility.

### Immunofluorescence assays

Immunofluorescence assays (IFA) were performed as described [46], using primary antibodies as follows: rabbit anti-Ripr [1∶1000]; rat anti-HA (monoclonal CF10, Roche) [1∶50]; rabbit anti-RON4 [44] [1∶250]; mouse anti-RAP1 [65] [1∶500]; mouse anti-Rh5 2F1 [1∶200]; rabbit anti-EBA175 [66] [1∶300]. Images were captured on a Zeiss Axiovert 200 m microscope (Zeiss) with a 100x/1.40NA PlanApochromat phase contrast oil immersion objective lens (Zeiss), an Axiocam MRm camera (Zeiss) and running Axiovision version 4.8 software (Zeiss).

### Differential solubilization of membrane proteins

PfRiprHA parasite (late schitzont stage)-infected red blood cells were hypotonically lysed with water, centrifuged and the pellet fraction washed with PBS. The pellet was then divided into four eppendorf tubes and incubated on ice for 2 hr with 10 mM Tris/pH 8.0; 100 mM sodium carbonate/pH 11.5; 2% Triton X100 and 2% CHAPS in 50 mM Tris/pH8.0 containing 1 mM EDTA and 100 mM sodium chloride respectively. The samples were then centrifuged to separate soluble and insoluble fractions. The insoluble fraction was washed twice with PBS and analyzed by Western blot together with the soluble fraction. Saponin pellet, prepared from the late schizont stage PfRiprHA parasites, were subjected to the same analyses as described above.

### Growth inhibition assay (GIA)

GIA were performed as described [67]. Briefly, late trophozoite stage parasites were added to erythrocytes to give a parasitemia of 0.2% and haematocrit of 2% in 45 µl of 0.5% Albumax II (Gibco, Auckland, New Zealand) in 96 well round bottom microtiter plates (Becton Dickinson, Fanklin Lakes, NJ, U.S.A.). 5 µl of purified rabbit IgG was added to a final concentration of 2 mg/ml. For the titration experiments, the antibodies were added to a final concentration of 0.125, 0.25, 0.5, 1, 2.0 and 3.0 mg/ml. Cultures were incubated for 72 hr (2 cycles) of growth and parasitaemia of each well was counted by flow cytometry, a FACSCalibur with a plate reader (Becton Dickinson, Fanklin Lakes, NJ, U.S.A.) after ethidium bromide (10 µg/ml, Biorad, Hercules, CA, U.S.A) staining trophozoite stage parasites. For each well, more than 50,000 cells were counted, and all samples were tested in triplicate. Growth was expressed as a percentage of the parasitaemia obtained from the pre-immunization IgG control. Three independent assays were performed.

### Merozoite attachment assay

Merozoite attachment was performed using viable cells purified as described [45], [50]. Briefly, late shizonts of 3D7 and FCR3 (40–46 hr post invasion) were purified from culture by magnet separation (Macs Miltenyi Biotec) to >95% purity. The purified schizonts were incubated with 10 µM E64 (Sigma) for 7–8 hr, then pelleted at 1900g for 5 min. Resulting parasitophorous vacuole membrane enclosed merozoites (PEMS) [68], [69] were resuspended in a small volume of incomplete culture medium (containing no protein) and filtered through a 1.2 µm, 32 mm syringe filter (Sartorius Stedim biotech, France). Antibodies were added to filtered merozoites at a final concentration of 2 mg/ml and incubated for 2 min after which uninfected erythrocytes were added and incubated for 10 min. Following fixation at room temperature for 30 min using 0.0075% glutaraldehyde/4% paraformaldehyde (ProSciTech, Australia) in PBS [70] the merozoite-bound erythrocytes were washed and stained with 0.1 ng/µL 4′,6-diamidino-2-phenylindole (DAPI) (Invitrogen). Imaging was performed using a either Plan-Apochromat 100x/1.40NA or 40x/1.3NA oil immersion Phase contrast lens (Zeiss) on an AxioVert 200 M microscope (Zeiss) equipped with an AxioCam Mrm camera (Zeiss). The MosaiX application from the Axiovision release 4.8 software (Zeiss) was used for counting. For each condition, at least 2000 red blood cells with bound merozoites were scored.

### Immuno-Electron microscopy

Merozoites from 3D7RiprHA parsites were purified according to a previously described method [50] and mixed with uninfected human erythrocytes. After a 2 min incubation, invading merozoites were fixed in 1% glutaraldehyde (Pro SciTech, Australia) in RPMI Hepes for 30 min on ice and prepared for Transmission electron microscopy as described [71]. Briefly, samples were dehydrated with increasing ethanol concentrations and embedded in LR Gold resin (Pro SciTech, Australia). The resin was polymerised using benzoyl peroxide (0.5%) (SPI-Chem, USA) and the preparation was sectioned on a Leica Ultracut R ultramicrotome (Wetzlar). The sections were blocked with 5% BSA and 0.1% Tween-20 in PBS for 30 min. Immunolabeling was performed with mouse anti-Rh5 clone 6H2 (dilution 1∶100) and mouse anti-HA clone 12AC5 (dilution 1∶100). Samples were incubated with 18 nm colloidal gold-conjugated goat anti-mouse secondary antibody (Jackson ImmunoResearch, Baltimore, USA). Post-staining was done with 2% aqueous uranyl acetate and 5% triple lead and observed at 120 kV on a Philips CM120 BioTWIN Transmission Electron Microscope.

## Supporting Information

Figure S1 **Elution profile of protein standards (BioRad).** The protein standards were analysed on the same Superdex 200 analytical column under the same conditions used for analysing PfRh5 complex shown in Figure 1A.
(TIF)

Figure S2 **Subcellular localization of PfRipr by immuno-electron microscopy.** Additional examples of *P. falciparum* 3D7RiprHA merozoites labelled with a primary HA antibody (mouse 12AC5) and a secondary goat-anti-mouse antibody conjugated to 18 nm colloidal gold. HA-tagged PfRipr was localised to the periphery of the parasite (possibly the surface) and to the interior of the apex of the parasite. Labelling is not observed inside rhoptries, but some PfRipr is localised adjacent to rhoptries (A, C), sometimes visible in electron dense bodies near the parasite apex consistent with micronemes (e.g. panel E), as well as being distributed around the periphery of free and invading merozoites. Panel (A) shows a merozoite in the process of invasion. Panels (B-F) show free merozoites – we are unable to determine which are more recently released from schizonts, and which are more mature. Scale bars show 200 nm.
(TIF)

Figure S3 **Antibodies to a N-terminal region of PfRipr inhibit parasite growth**. (A) Anti-PfRipr/3 antibodies inhibit invasion of *P. falciparum* strains into erythrocytes. Shown are growth inhibition assays of the parasite strains FCR3, W2mef, T994, CSL2, E8B, MCAMP, 7G8, D10, HB3 and 3D7. The final antibody concentration is 2 mg/ml. (B) Titration of anti-PfRipr/3 antibodies in growth inhibition assays of the 3D7 strain.
(TIF)

Figure S4 **Anti-PfRipr antibodies inhibit the parasite growth of a sialic acid-dependent parasite strain (W2mf) more effectively than a sialic acid-independent strain (W2mefΔ175).**
(TIF)

Figure S5 **PfRh5/PfRipr complex might dissociate upon PfRh5 binding to erythrocytes.** (A) Purification of PfRh5/PfRipr complex from culture supernatant of 3D7PfRiprHA parasites by an ion-exchange column. The NaCl eluted fractions were probed for PfRh5 and PfRipr. (B) Red blood cell binding assay using PfRh5/PfRipr complex (#6) partially purified from culture supernatant of 3D7PfRiprHA parasites by the ion-exchange column. Analyses of eluted fraction detect PfRh5 but not PfRipr, indicating that PfRh5/PfRipr complex might dissociate upon PfRh5 binding to erythrocytes.
(TIF)

Figure S6 **Polymorphisms of PfRipr protein in** ***P. falciparum*** **strains.** Only positions of amino acid changes were indicated. Amino acid sequences 238–368 and 791–900 were used to make recombinant proteins for antibody production.
(TIF)

Figure S7 **Additive inhibition of anti-PfRipr/1 in combination with antibodies to EBA-175 or/and PfRh4 for the FCR3 strain of** ***P. falciparum*** **.** The final antibody concentration is 1 mg/ml. In all the cases, each graph represents three independent experiments done in triplicate with each normalised to the negative control (Protein A purified IgG from normal rabbit serum). The error bars represent standard error of the mean of the three independent experiments.
(TIF)

Figure S8 **Strategy used for the attempted disruption of the** ***PFC1045c*** **gene in** ***P. falciparum*** **using the plasmid vector pCC1.** The *hdhfr* cassette would be inserted by homologous double crossover recombination between the 5′ and 3′ *PFC1045c* flanks (black shaded boxes) in the vector and the endogenous locus. Restriction sites are shown, *Bam*H1, B; *Nco* 1, N; *Eco*R1, E; *Afl* II, A. WR, WR99210. FC, 5′ fluoro-cytosine. The sizes of the bands expected in Southern blot experiments are shown in kilobase pairs (kb). The bottom panels are Southern blots to confirm that integration of the transfected episome had not happened. The bands in the first panel represent the episomal plasmid (9 kb) and the intact *PFC1045c* gene (1 kb). In the 3D7 untransfected line the episomal band is absent as expected; however, after two cycles both the intact gene and plasmid bands are obtained when probed with the 5′ flank. The second panel represents a second independent transfection in which no integration of the transfected pCC1 vector was observed.
(TIF)

Figure S9 **PCR analysis of the attempted disruption of the** ***PFC1045c*** **gene in** ***P. falciparum*** **using the plasmid vector pCC1.** The *hdhfr* cassette would be inserted by homologous double crossover recombination between the 5′ and 3′ *PFC1045c* flanks (black shaded boxes) in the vector and the endogenous locus. PCR analysis of genomic DNA from 3D7 transfected with pCC1-PFC1045c that confers resistance to WR99210 and sensitivity to 5-Fluro-cytosine. For 3D7 the endogenous gene was detected with p405/p338 oligonucleotide primers (1454 bp), whilst for 3D7ΔPFC1045c the PCR product if present would be detected with p403/p560 (1373 bp), and this would represent integration of the *hdhfr* gene and disruption of *PFC1045c*. In the case of 3D7 transfected with pCC1-PFC1045c a PCR product was observed for wild type, but not for the event corresponding to integration by homologous recombination. PCR oligonucleotide pairs p403/p338 and p335/p388 were used as specificity controls. Both PCR oligonucleotide pairs produced PCR products of the expected size.
(TIF)

Table S1 **Mass spectrometry identification of PfRh5 complex.** A list of all proteins identified by mass spectrometry from the PfRh5 purified complex.
(DOC)
