# Supplementary figures and images for: An EGF-like Protein Forms a Complex with PfRh5 and Is Required for Invasion of Human Erythrocytes by Plasmodium falciparum
Source: PLoS Pathog. 2011 Sep 1;7(9):e1002199. doi: 10.1371/journal.ppat.1002199 (PMC3164636; doi:10.1371/journal.ppat.1002199)

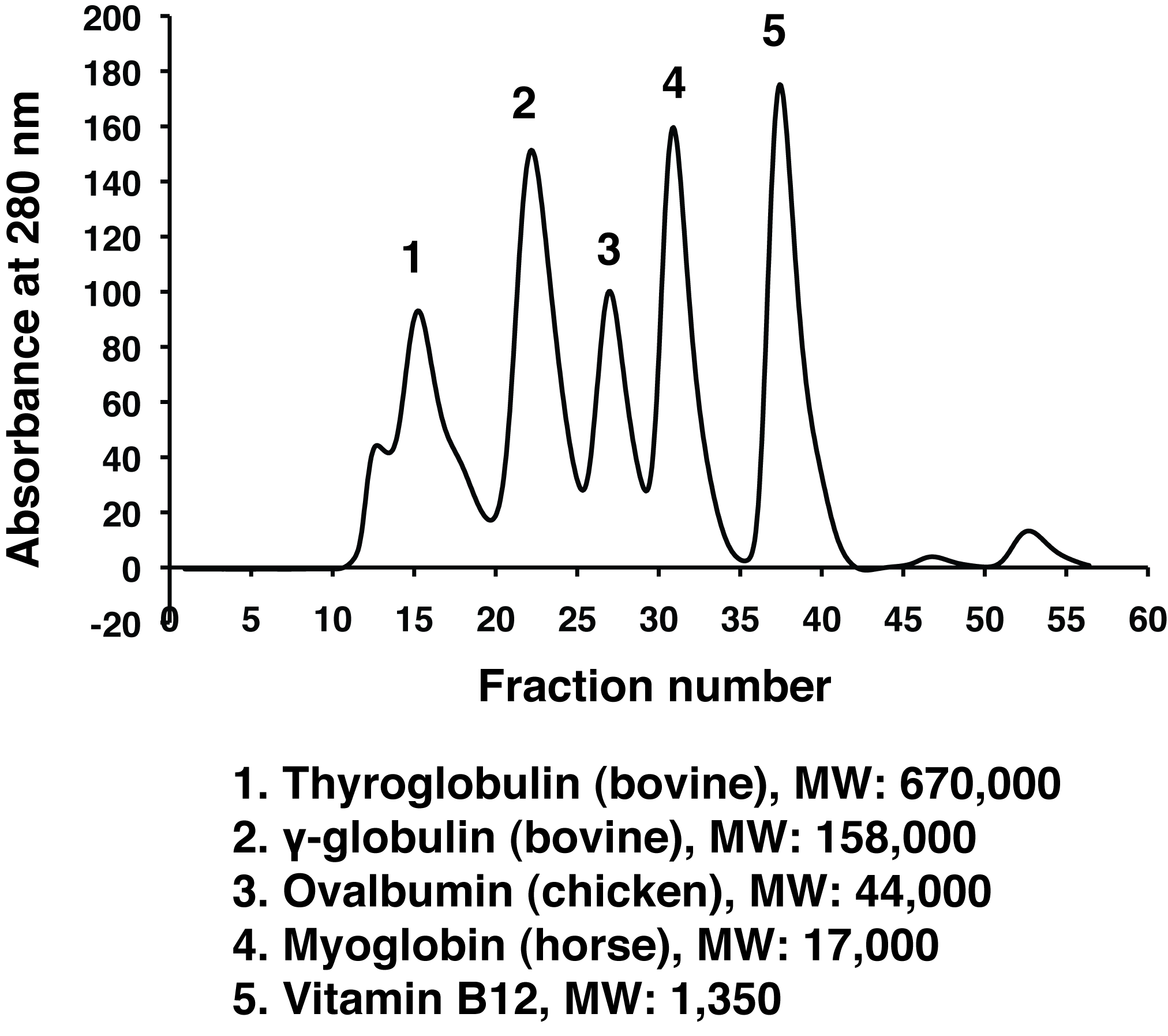

Supplement: Figure S1 — Elution profile of protein standards (BioRad). The protein standards were analysed on the same Superdex 200 analytical column under the same conditions used for analysing PfRh5 complex shown in Figure 1A. (TIF) [file ppat.1002199.s001.tif]

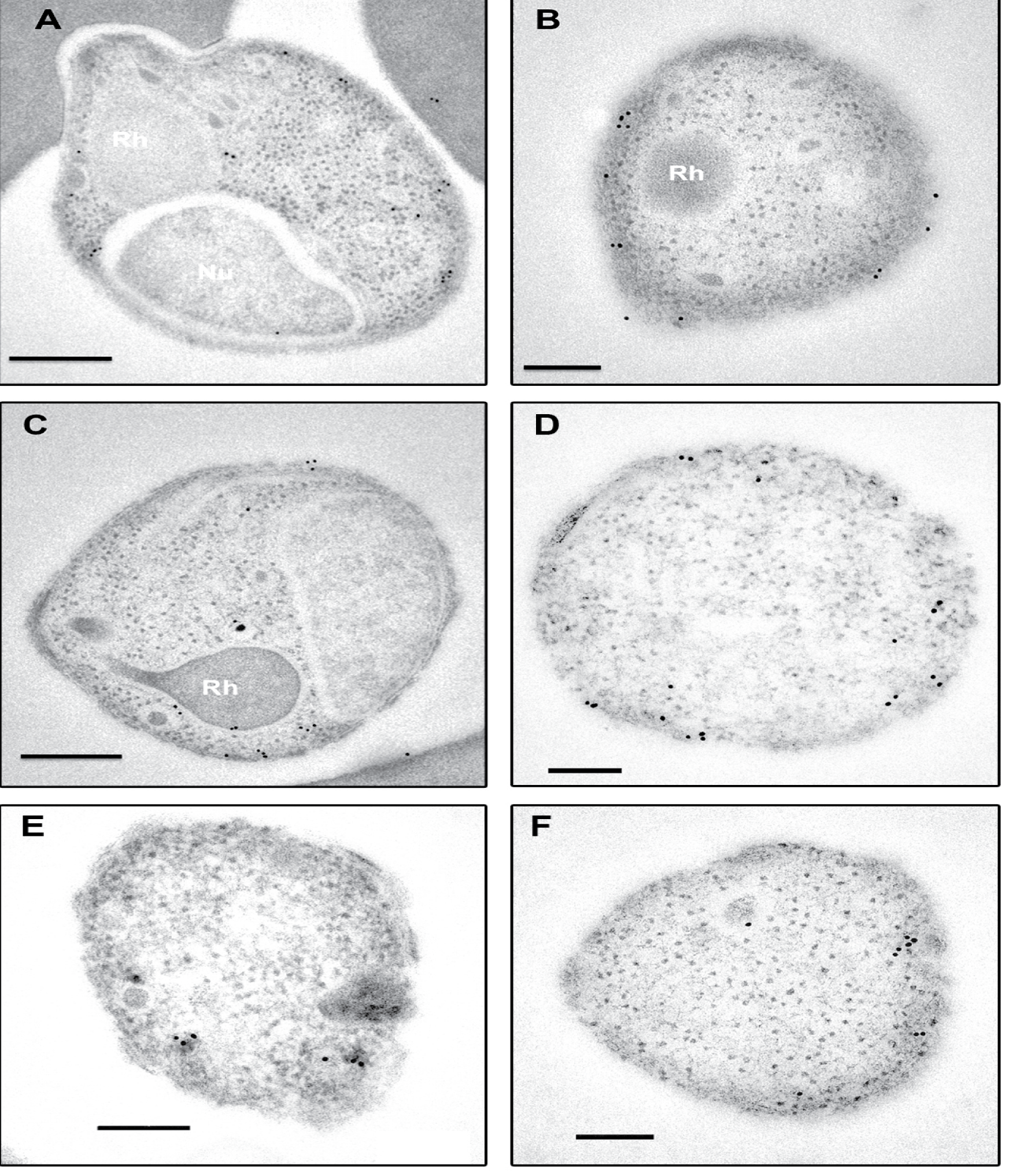

Supplement: Figure S2 — Subcellular localization of PfRipr by immuno-electron microscopy. Additional examples of P. falciparum 3D7RiprHA merozoites labelled with a primary HA antibody (mouse 12AC5) and a secondary goat-anti-mouse antibody conjugated to 18 nm colloidal gold. HA-tagged PfRipr was localised to the periphery of the parasite (possibly the surface) and to the interior of the apex of the parasite. Labelling is not observed inside rhoptries, but some PfRipr is localised adjacent to rhoptries (A, C), sometimes visible in electron dense bodies near the parasite apex consistent with micronemes (e.g. panel E), as well as being distributed around the periphery of free and invading merozoites. Panel (A) shows a merozoite in the process of invasion. Panels (B-F) show free merozoites – we are unable to determine which are more recently released from schizonts, and which are more mature. Scale bars show 200 nm. (TIF) [file ppat.1002199.s002.tif]

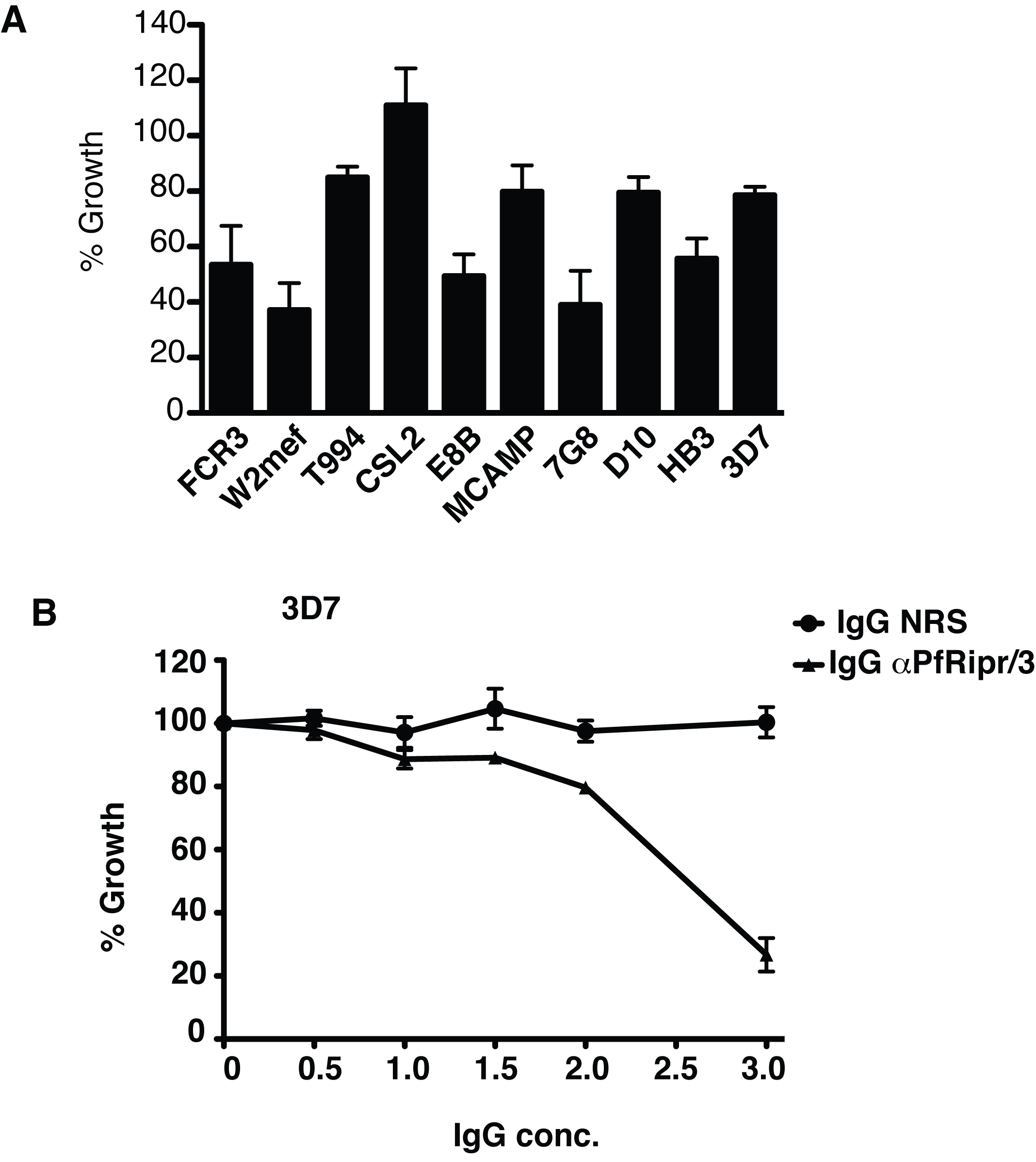

Supplement: Figure S3 — Antibodies to a N-terminal region of PfRipr inhibit parasite growth. (A) Anti-PfRipr/3 antibodies inhibit invasion of P. falciparum strains into erythrocytes. Shown are growth inhibition assays of the parasite strains FCR3, W2mef, T994, CSL2, E8B, MCAMP, 7G8, D10, HB3 and 3D7. The final antibody concentration is 2 mg/ml. (B) Titration of anti-PfRipr/3 antibodies in growth inhibition assays of the 3D7 strain. (TIF) [file ppat.1002199.s003.tif]

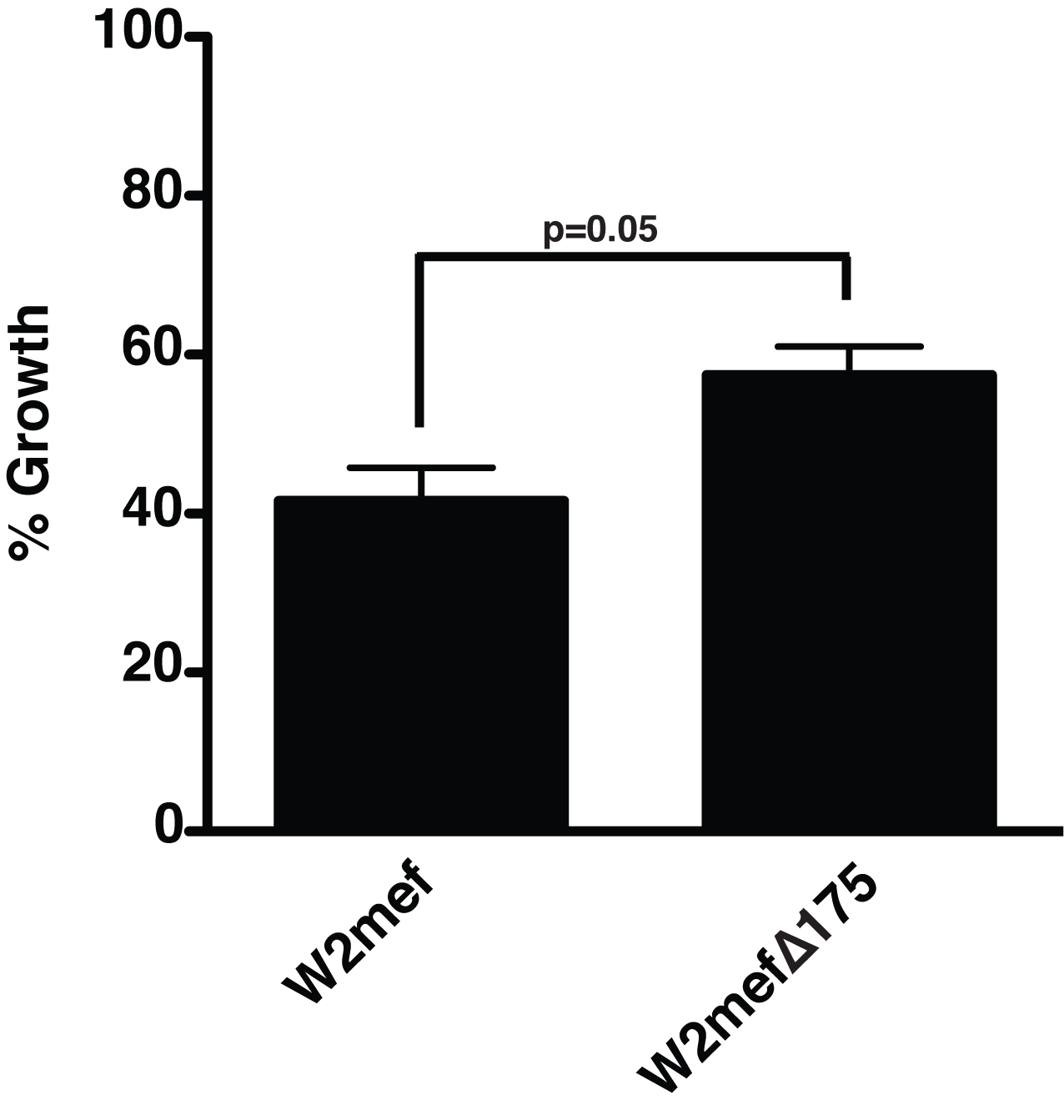

Supplement: Figure S4 — Anti-PfRipr antibodies inhibit the parasite growth of a sialic acid-dependent parasite strain (W2mf) more effectively than a sialic acid-independent strain (W2mefΔ175). (TIF) [file ppat.1002199.s004.tif]

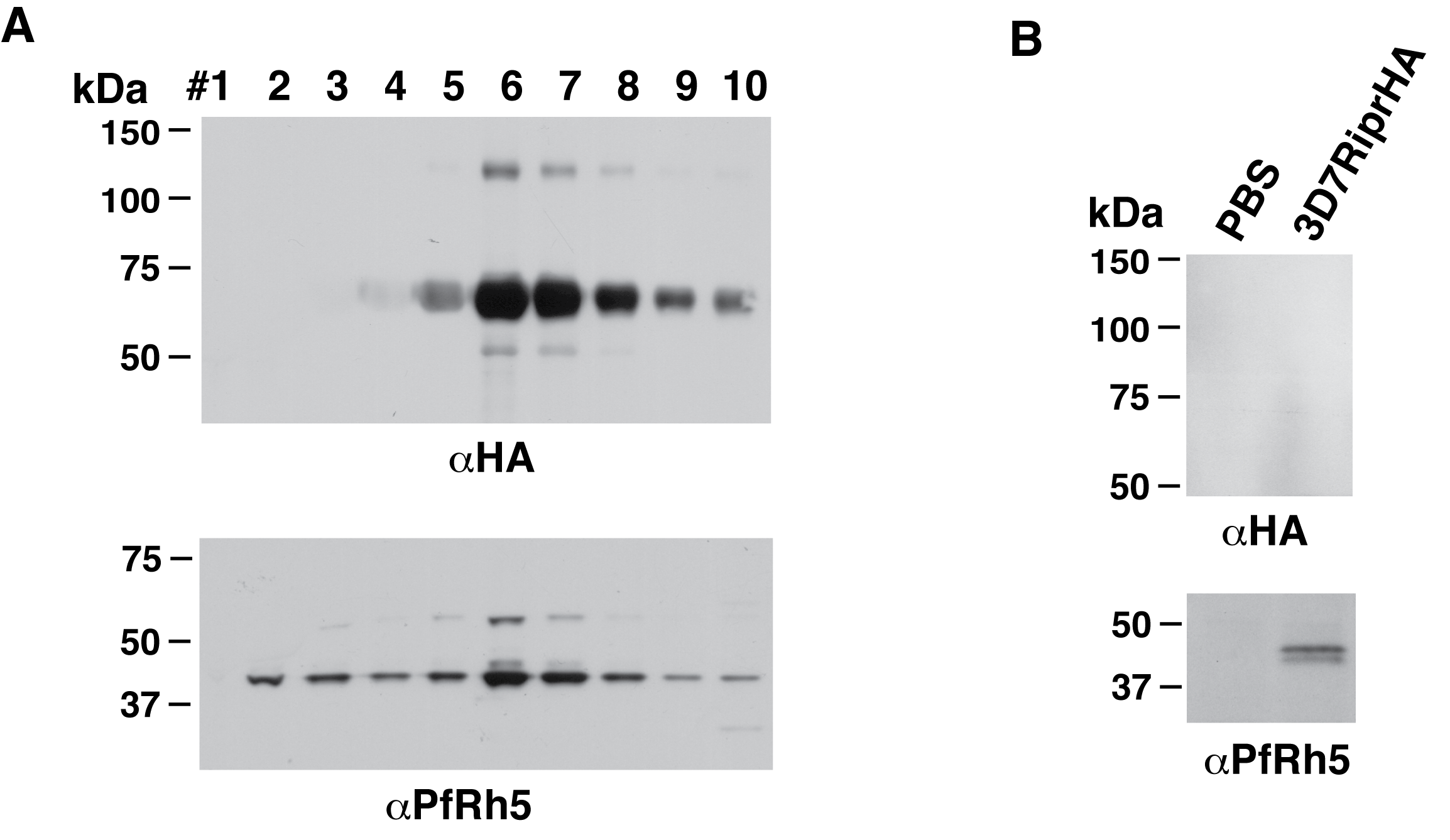

Supplement: Figure S5 — PfRh5/PfRipr complex might dissociate upon PfRh5 binding to erythrocytes. (A) Purification of PfRh5/PfRipr complex from culture supernatant of 3D7PfRiprHA parasites by an ion-exchange column. The NaCl eluted fractions were probed for PfRh5 and PfRipr. (B) Red blood cell binding assay using PfRh5/PfRipr complex (#6) partially purified from culture supernatant of 3D7PfRiprHA parasites by the ion-exchange column. Analyses of eluted fraction detect PfRh5 but not PfRipr, indicating that PfRh5/PfRipr complex might dissociate upon PfRh5 binding to erythrocytes. (TIF) [file ppat.1002199.s005.tif]

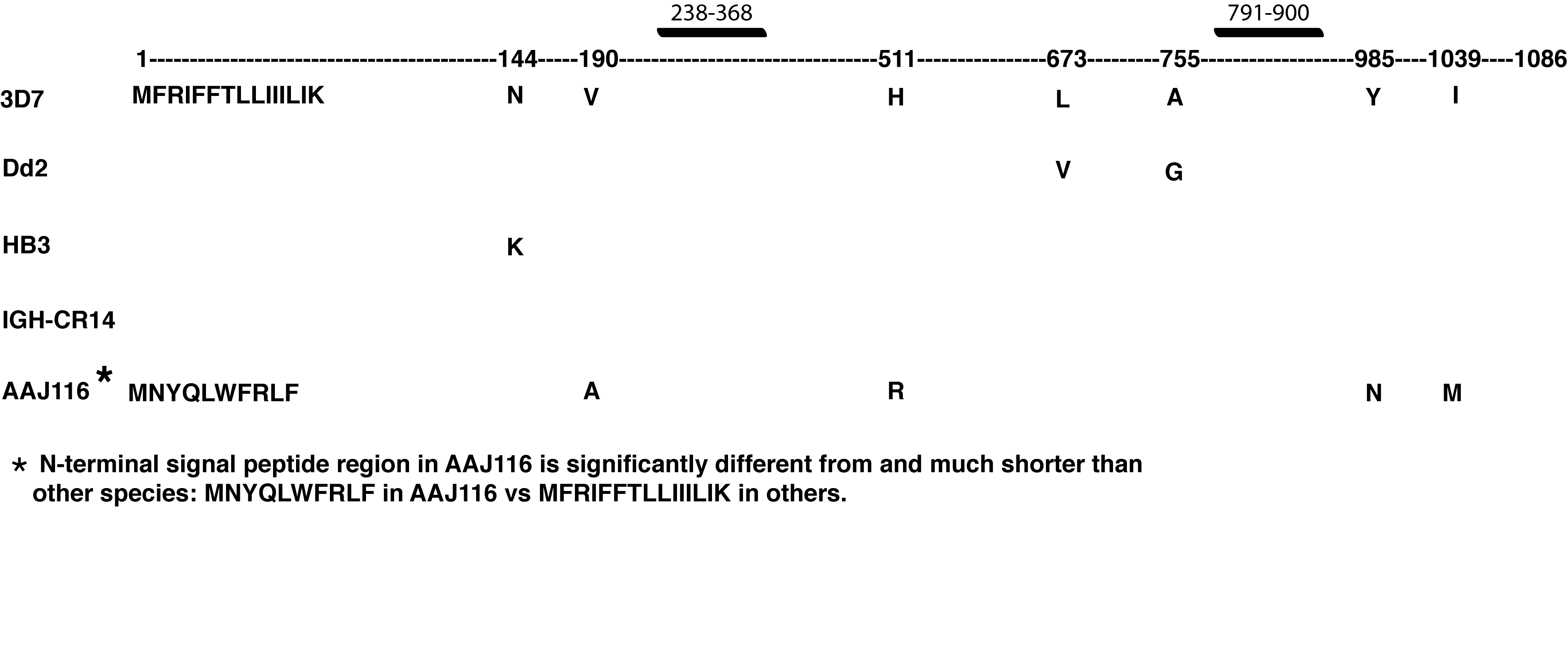

Supplement: Figure S6 — Polymorphisms of PfRipr protein in P. falciparum strains. Only positions of amino acid changes were indicated. Amino acid sequences 238–368 and 791–900 were used to make recombinant proteins for antibody production. (TIF) [file ppat.1002199.s006.tif]

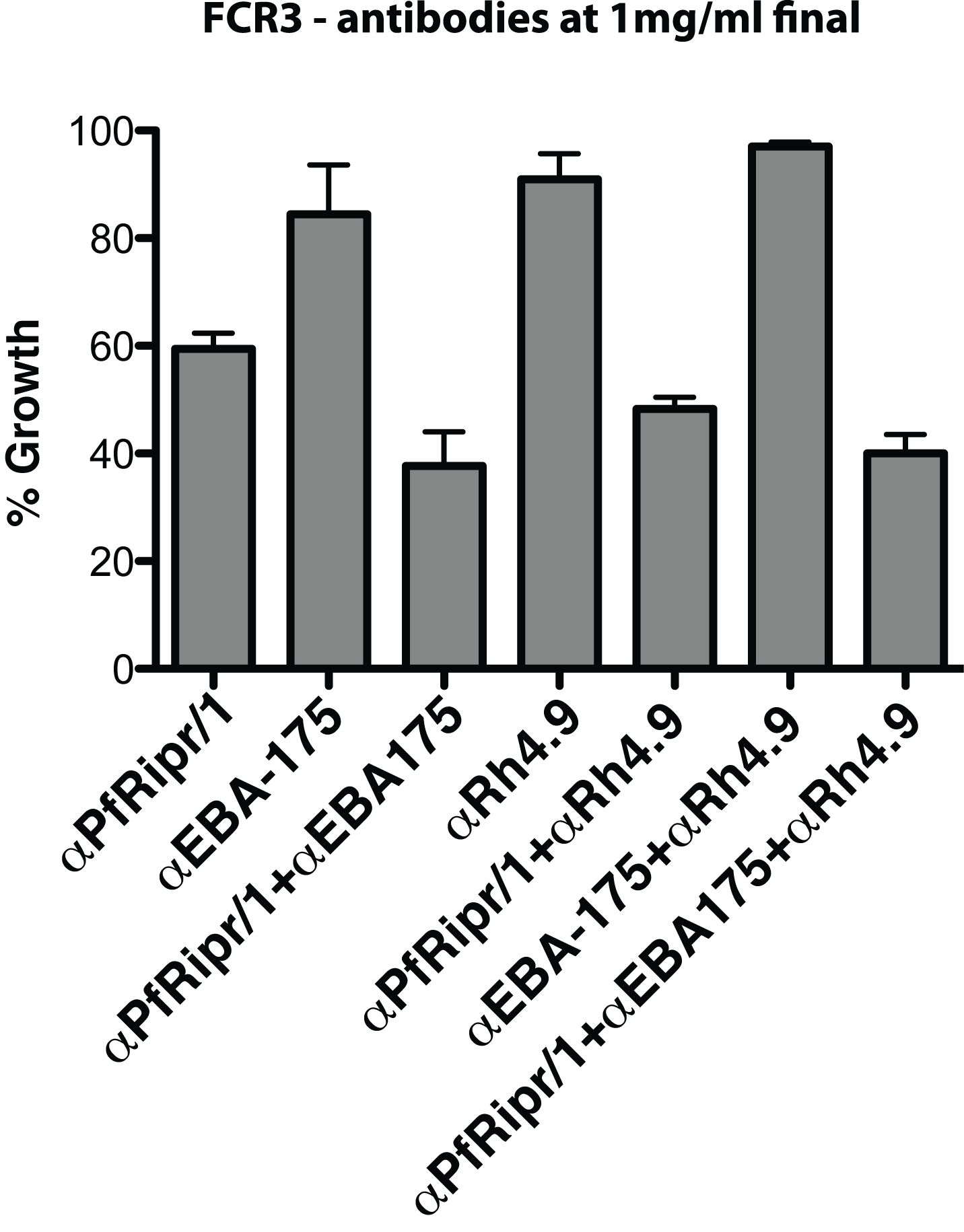

Supplement: Figure S7 — Additive inhibition of anti-PfRipr/1 in combination with antibodies to EBA-175 or/and PfRh4 for the FCR3 strain of P. falciparum . The final antibody concentration is 1 mg/ml. In all the cases, each graph represents three independent experiments done in triplicate with each normalised to the negative control (Protein A purified IgG from normal rabbit serum). The error bars represent standard error of the mean of the three independent experiments. (TIF) [file ppat.1002199.s007.tif]

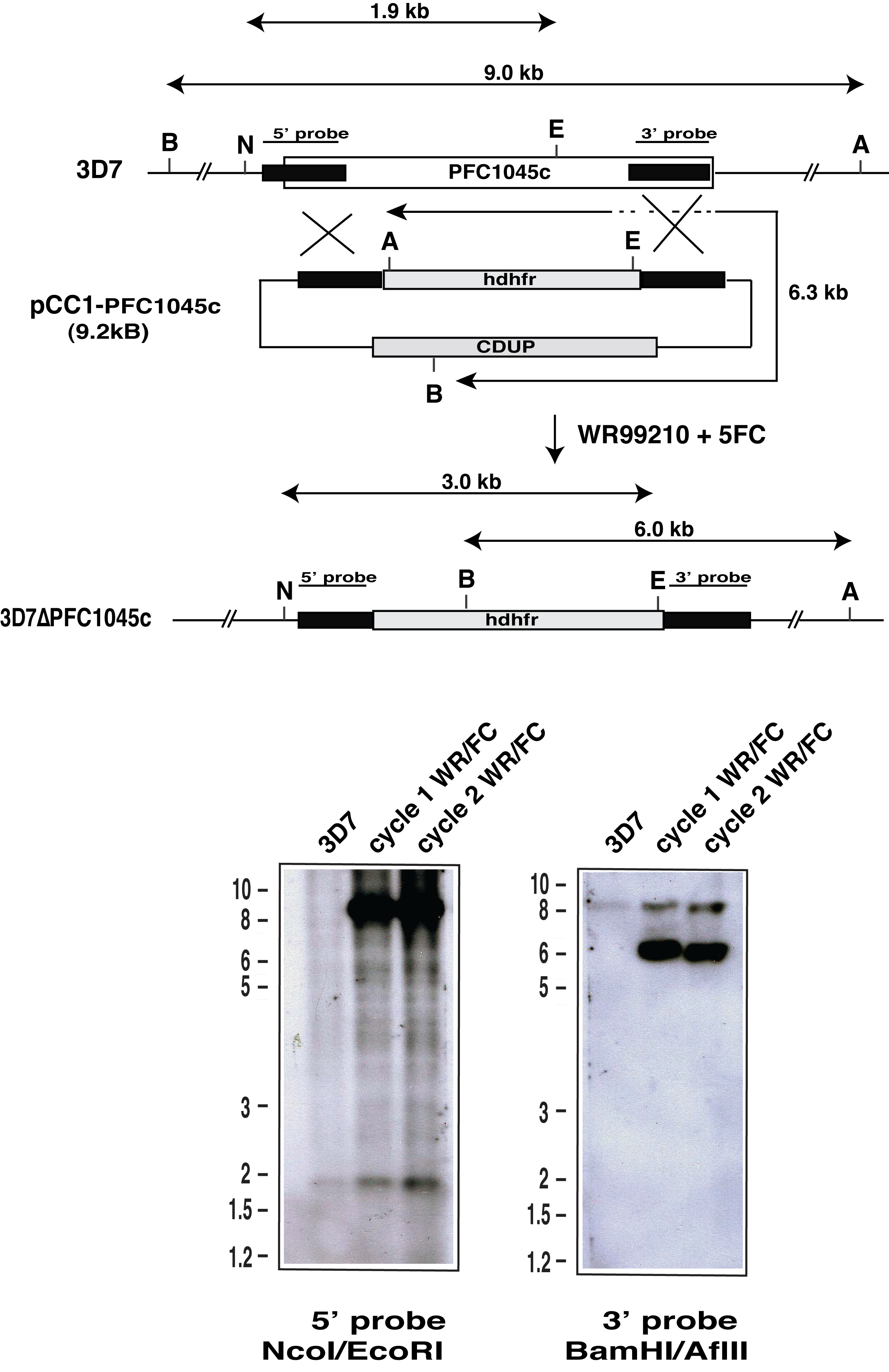

Supplement: Figure S8 — Strategy used for the attempted disruption of the PFC1045c gene in P. falciparum using the plasmid vector pCC1. The hdhfr cassette would be inserted by homologous double crossover recombination between the 5′ and 3′ PFC1045c flanks (black shaded boxes) in the vector and the endogenous locus. Restriction sites are shown, BamH1, B; Nco 1, N; EcoR1, E; Afl II, A. WR, WR99210. FC, 5′ fluoro-cytosine. The sizes of the bands expected in Southern blot experiments are shown in kilobase pairs (kb). The bottom panels are Southern blots to confirm that integration of the transfected episome had not happened. The bands in the first panel represent the episomal plasmid (9 kb) and the intact PFC1045c gene (1 kb). In the 3D7 untransfected line the episomal band is absent as expected; however, after two cycles both the intact gene and plasmid bands are obtained when probed with the 5′ flank. The second panel represents a second independent transfection in which no integration of the transfected pCC1 vector was observed. (TIF) [file ppat.1002199.s008.tif]

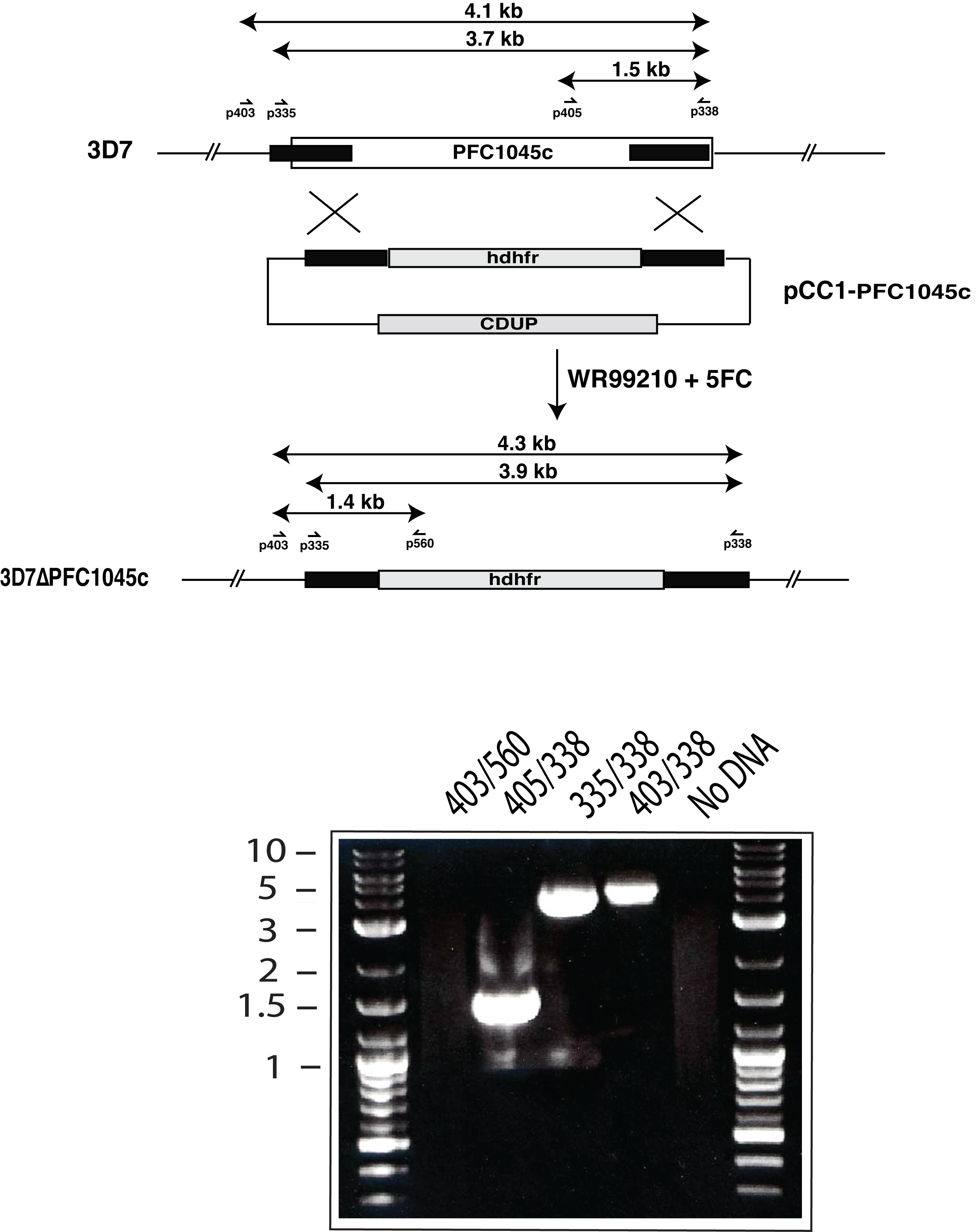

Supplement: Figure S9 — PCR analysis of the attempted disruption of the PFC1045c gene in P. falciparum using the plasmid vector pCC1. The hdhfr cassette would be inserted by homologous double crossover recombination between the 5′ and 3′ PFC1045c flanks (black shaded boxes) in the vector and the endogenous locus. PCR analysis of genomic DNA from 3D7 transfected with pCC1-PFC1045c that confers resistance to WR99210 and sensitivity to 5-Fluro-cytosine. For 3D7 the endogenous gene was detected with p405/p338 oligonucleotide primers (1454 bp), whilst for 3D7ΔPFC1045c the PCR product if present would be detected with p403/p560 (1373 bp), and this would represent integration of the hdhfr gene and disruption of PFC1045c. In the case of 3D7 transfected with pCC1-PFC1045c a PCR product was observed for wild type, but not for the event corresponding to integration by homologous recombination. PCR oligonucleotide pairs p403/p338 and p335/p388 were used as specificity controls. Both PCR oligonucleotide pairs produced PCR products of the expected size. (TIF) [file ppat.1002199.s009.tif]
