# Supplementary material for: An EGF-like Protein Forms a Complex with PfRh5 and Is Required for Invasion of Human Erythrocytes by Plasmodium falciparum
Source: PLoS Pathog. 2011 Sep 1;7(9):e1002199. doi: 10.1371/journal.ppat.1002199 (PMC3164636; doi:10.1371/journal.ppat.1002199)
Supplement: Table S1 — Mass spectrometry identification of PfRh5 complex. A list of all proteins identified by mass spectrometry from the PfRh5 purified complex. (DOC) [file ppat.1002199.s010.doc]

Supplementary table1: Mass spectrometry identification of PfRh5 complex:

| Protein name | Number of peptide identified | Accession number |
| --- | --- | --- |
| PfRh5 [Plasmodium falciparum] | 6 | Q8IFM5 |
| Putative uncharacterized protein [Plasmodium falciparum] --PfRipr | 5 | O97302 |
| Elongation factor 1-alpha [Plasmodium falciparum] | 2 | Q8I0P6 |
| Heat-shock protein [Plasmodium falciparum] | 1 | Q25869 |
| Keratin, type II cytoskeletal 1 [Homo sapiens] | 14 | P04264 |
| Keratin, type II cytoskeletal 2 epidermal [Homo sapiens] | 12 | P35908 |
| Keratin, type I cytoskeletal 9 [Homo sapiens] | 7 | P35527 |
| Keratin, type I cytoskeletal 10 [Homo sapiens] | 4 | PI3645 |
| Hemoglobin subunit alpha [Homo sapiens] | 2 | P69905 |
| Hemoglobin subunit beta [Homo sapiens] | 3 | P68871 |
| Hornerin [Homo sapiens] | 1 | Q5DT20 |
| Palmitoyl-protein thioesterase 1 [Homo sapiens] | 1 | P50897 |
| Serum albumin [Bos Taurus] | 5 | P02769 |
| IGHM protein [Bos Taurus] | 1 | QIRMK2 |
| Isoaprotinin G2 Bos Taurus] | 1 | Q7M312 |
| Pancreatic elastase inhibitor [Bos Taurus] | 1 | Q9TS74 |
